# Exploration of factors affecting webcam-based automated gaze coding

**DOI:** 10.3758/s13428-024-02424-1

**Published:** 2024-05-01

**Authors:** Hiromichi Hagihara, Lorijn Zaadnoordijk, Rhodri Cusack, Nanako Kimura, Sho Tsuji

**Affiliations:** 1 Graduate School of Human Sciences, Osaka University 1-2 Yamadaoka, Suita-shi Osaka, 565-0871 Japan; 2 International Research Center for Neurointelligence (WPI-IRCN), The University of Tokyo Institutes for Advanced Study, 7-3-1 Hongo, Bunkyo-ku, Tokyo, 113-0033 Japan; 3 Japan Society for the Promotion of Science, 5-3-1 Kojimachi, Chiyoda-ku, Tokyo, 102-0083 Japan; 4 The Institute for AI and Beyond, The University of Tokyo, 2-11-16, Yayoi, Bunkyo-ku, Tokyo, 113-0032 Japan; 5 Trinity College Institute of Neuroscience and School of Psychology, Trinity College Dublin, College Green, Dublin 2, Ireland; 6 Graduate School of Engineering, The University of Tokyo, 7-3-1 Hongo, Bunkyo-ku, Tokyo, 113-8656 Japan

**Keywords:** Online experiment, Webcam video data, Automated gaze coding, Data quality, Open dataset

## Abstract

Online experiments have been transforming the field of behavioral research, enabling researchers to increase sample sizes, access diverse populations, lower the costs of data collection, and promote reproducibility. The field of developmental psychology increasingly exploits such online testing approaches. Since infants cannot give explicit behavioral responses, one key outcome measure is infants’ gaze behavior. In the absence of automated eyetrackers in participants’ homes, automatic gaze classification from webcam data would make it possible to avoid painstaking manual coding. However, the lack of a controlled experimental environment may lead to various noise factors impeding automatic face detection or gaze classification. We created an adult webcam dataset that systematically reproduced noise factors from infant webcam studies which might affect automated gaze coding accuracy. We varied participants’ left-right offset, distance to the camera, facial rotation, and the direction of the lighting source. Running two state-of-the-art classification algorithms (iCatcher+ and OWLET) revealed that facial detection performance was particularly affected by the lighting source, while gaze coding accuracy was consistently affected by the distance to the camera and lighting source. Morphing participants’ faces to be unidentifiable did not generally affect the results, suggesting facial anonymization could be used when making online video data publicly available, for purposes of further study and transparency. Our findings will guide improving study design for infant and adult participants during online experiments. Moreover, training algorithms using our dataset will allow researchers to improve robustness and allow developmental psychologists to leverage online testing more efficiently.

**Supplementary Information:**

The online version contains supplementary material available at 10.3758/s13428-024-02424-1.

## Introduction

Online experiments have enabled psychologists to collect data efficiently, irrespective of participants’ locations (Semmelmann & Weigelt, 2018; Tran et al., 2017; Zaadnoordijk et al., 2021; Zaadnoordijk & Cusack, 2022). In a typical online experiment, participants log in to an online experimental platform with their digital device and respond to prompts on screen like they would in a lab experiment. Often, this is possible in asynchronous fashion (e.g., via the browser), meaning participants can participate at a time convenient for them without making an appointment. This methodological innovation potentially facilitates robust science, on the one hand by enlarging sample sizes due to the decreased time and financial costs. Furthermore, it can help to access more diverse populations when it comes to ethnicity, language, or socioeconomic status due to the lack of geographical restrictions on participation. Online experiments may therefore contribute to overcoming pressing concerns in the field of psychology such as the lack of reproducibility (Open Science Collaboration, 2015) and the dominance of culturally biased samples (Henrich et al., 2010).

In recent years, researchers focusing on child development have increasingly used online experiments. The notable advancement of online methods in this field can be seen through the creation of platforms designed for infant behavioral research (e.g., Lo et al., 2021; Scott & Schulz, 2017), international collaborative initiatives (e.g., Zaadnoordijk et al., 2021), guides for researchers (Kominsky et al., 2021; Rhodes et al., 2020, Zaadnoordijk & Cusack, 2022), and special journal issues on the topic (e.g., Tsuji et al., 2022). Online experiments are attractive to developmental psychologists, not only because the spread of COVID-19 made in-person experiments difficult, but also because the problems of small sample sizes and lack of diversity are potentially exacerbated in participants recruited into labs.

Experimental studies on young children often have low statistical power due to small sample sizes and increased measurement noises (Bergmann et al., 2018; Byers-Heinlein et al., 2022; Davis-Kean & Ellis, 2019; DeBolt et al., 2020; Oakes, 2017). One major reason is that bringing young children to the lab is an effortful process for all parties involved. Online testing may alleviate this because it is convenient for both researchers and participants. Researchers can run online experiments with lower costs in terms of time and money compared with in-lab experiments. Dozens of participants can be recruited for online experiments in a single day (Berinsky et al., 2012; Casler et al., 2013; Tran et al., 2017). Parents can participate in studies at a time that is convenient for them and their children. Children are likely to be calmer and more patient in their familiar home environment. These advantages potentially increase the possibilities of successful data collection and reduce dropouts. Another concern researchers have faced is biased samples, with most psychological studies being conducted on globally unrepresentative WEIRD (Western, Educated, Industrialized, Rich, and Democratic) populations (Henrich et al., 2010; Singh et al., 2021). Because researchers can recruit anyone who has a computer connected to the Internet for online experiments, they have the potential to reach more diverse populations who cannot easily access in in-lab experiments, ranging from participants living in countries with no developmental labs to those in rural areas who live far from labs or those who work full time and cannot make daytime testing sessions (Bacon et al., 2021; Rhodes et al., 2020; Scott & Schulz, 2017; Zaadnoordijk & Cusack, 2022; but see Lourenco & Tasimi, 2020). Moreover, online testing may even improve the replicability and transparency of experimental protocols due to the absence of a human experimenter during asynchronous online testing (Zaadnoordijk et al., 2021). Researchers have to specify in detail and automate the experimental design, protocol, and instructions. This reduces the possibility for researchers’ implicit flexible decisions to vary across participants and potentially makes the replication of the same experiment easier and more reliable.

A remaining major cost factor and potential source of subjectivity in online developmental studies is the need to manually tag the infant’s gaze direction in the recorded video. Gaze data is the main outcome measure in many experimental lab studies targeting infants and young children, because they often cannot yet give explicit verbal or behavioral responses, and because looking behavior can reflect children’s attention, preference, and choices (Hagihara et al., 2021). Gaze-based measures have been leveraged for a long time in various domains in the field of developmental science (e.g., Aslin 2007; Fantz, 1964; Starkey et al., 1983). In such studies, infants are typically seated in front of a computer screen which displays visual and/or auditory stimuli while their gaze behavior is monitored. In most cases, coarse gaze coding suffices for infant studies: Their gaze is either classified into two categories that divide gaze into those on or off the screen (“look” and “away”; Hamlin et al., 2007; Maye et al., 2002; Montague & Walker-Andrews, 2001). This kind of coding is, for instance, used to measure infants’ degree of attention to the displayed stimuli. Slightly more detailed is a coding scheme into three categories of looking “left,” “right,” or “away” (Bailey & Plunkett, 2002; Fernald et al., 1998, 2008; Golinkoff et al., 1987, 2013; Yuan & Fisher, 2009). This kind of scheme can be used to assess infants’ choice of one over another stimulus, for instance if two visual stimuli are displayed concurrently to the left and right of the screen. Despite these simple coding schemes, frame-by-frame manual coding from video data is quite labor-intensive, requiring extensive training and taking at least several times as long as the actual video duration (Erel et al., 2022, 2023; Friend & Keplinger, 2008; Venker et al., 2020). Although automatic eye trackers have nowadays often replaced such manual coding in laboratory settings, online experiments cannot leverage such devices.

A potential solution to the challenge of manual coding is automatic gaze coding from a webcam. Good automatic gaze coding solutions now exist for adult participants (Papoutsaki et al., 2016; Zhang et al., 2019). However, the data resulting from online experiments for young children often entail various environmental or behavioral noise factors that makes it more difficult to classify gaze directions automatically. Here, we operationally defined “noise” as a group of factors lowering the data quality in ways that affect gaze coding performance. Such factors, for instance, affect precision, the degree to which one can reproduce a true gaze location as measured by the eye-tracking device, and data loss, the data points that would be expected to be measured but have not been measured (Hessels & Hooge, 2019). Different from adults, young children cannot yet be instructed to sit still facing the screen, and in the absence of a controlled experimental setup and a trained experimenter in at-home experiments, such noise factors can aggravate the quality of video data. For instance: infants’ faces are not always fully visible (Erel et al., 2023) or properly upright (Hessels, Cornelissen et al, 2015b; Niehorster et al., 2018); they are sometimes positioned on the left- or right-hand side of the webcam (Erel et al., 2022); and infants move their bodies during data collection (Dalrymple et al., 2018; Hessels, Andersson et al., 2015a; Schlegelmilch & Wertz, 2019; Wass et al., 2014). In addition, environmental factors such as screen size of the used device and lighting conditions might vary.

Although a recent meta-analysis reported compatible effect sizes between developmental in-lab and online experiments (Chuey et al., 2021), this meta-analysis only included moderated studies, that is, ones where an experimenter was present. However, many researchers have turned to asynchronous testing to maximize the efficiency of data collection, such that different participants can engage in the same experiment in parallel without the presence of an experimenter (Zaadnoordijk et al., 2021). In such unmoderated cases, environmental and/or behavioral noise is likely harder to control, deteriorating video data quality and subsequently affecting automatic gaze coding. By instructing participants to be careful about noise factors just before the online experiment, for instance using an information video, researchers can avoid such noise contamination. In fact, a recent study reported that instructions to caregivers enhanced the data quality for online testing to such an extent that it did not differ from in-lab testing concerning several noise factors such as lighting conditions (Bánki et al., 2022).

The current study aimed to elucidate to what extent such factors affect the accuracy of existing automated gaze classifiers. We chose to create an adult webcam dataset collected in the lab that systematically reproduces environmental or behavioral noise encountered in data from child online experiments, since only an adult in-lab experiment would allow us to systematically reproduce these factors in a well-controlled fashion. We first explored and classified frequently occurring noise factors from existing experimental child online gaze data (Hagihara et al., 2022). Based on this exploration, we selected four factors that are frequently observed and potentially controllable in online experiments: participants’ left-right offset, distance to the camera, facial rotation (roll), and the direction of the lighting source. Some other factors such as infants’ head movement or eye closing are hardly controllable, and other ones such as the webcam’s spatial resolution or facial occlusion can be implemented by post hoc editing of the video dataset (see supplementary Table S1). We collected webcam data while varying the selected factors independently, as our exploration suggested that they did not co-occur systematically. We also accounted for different screen sizes and ran two state-of-the-art gaze classification algorithms developed for infant studies: iCatcher+ (Erel et al., 2022, 2023) and OWLET (Werchan et al., 2022).

The present study contributes to facilitating developmental researchers’ access to automatic gaze coding in two ways. On the one hand, the results from the present study will allow researchers to prioritize improving on those noise factors during data collection that affect automatic gaze coding the most, for instance by providing targeted instructions for parents. On the other hand, the openly accessible webcam dataset will enable researchers to improve automated classification models. In tandem with the increased conducting of online testing, webcam-based gaze coding has become even more important. This is evident as many researchers have recently tackled relevant topics, such as evaluating the utility of existing automated gaze coding algorithms (Steffan et al., 2024; Valtakari et al., 2023). More broadly, the results of the present study will also be informative beyond the field of child development, for researchers conducting online studies with special populations or in settings with significant environmental noise.

In the spirit of open science and in order to improve automatic gaze coding algorithms, ideally everyone would be able to share their video data sets. However, because raw video data capturing participants’ faces cannot be fully anonymized and infants cannot provide consent by themselves, it is ethically challenging to make infant video data publicly available. This hinders transparency and reproducibility in online infant studies as well as any other infant studies whose conclusions rely on video data. A second aim of the present study was therefore to explore solutions to replace participants’ faces with non-existing ones using facial swapping techniques (e.g., Li et al., 2019; Nirkin et al., 2019). Specifically, it is unclear to what extent the automated gaze coding accuracy before and after such facial anonymizations is equivalent because swapping faces may destroy relevant visual features of the original data. To assess this, in a second analysis we applied a state-of-the-art facial anonymization technique (Deep Natural Anonymization; BrighterAI, n.d.) to our video dataset and compared automatic gaze code classification from raw data to classification from the anonymized data.

## Methods

### Participants

A total of 60 adult participants were recruited in Dublin, Ireland, and Tokyo, Japan (30 participants from each site; Mean age = 26.5, *SD* = 7.6; 32 females, 27 males, one other). An additional participant engaged in the experimental session but was later excluded due to inattention and experimental error. Eight participants wore glasses but were asked to take them off during the experiment. All of them could see the visual stimuli appropriately. Eighteen participants used contact lenses. Among the 60 participants, 47 agreed with making their video recordings publicly available (uploaded at https://osf.io/48zvh/?view_only=22a82bc40bab441589660168a48944c5). All participants provided written consent before taking part in the experiment. This study was approved by the School of Psychology Research Ethics Committee, Trinity College Dublin (SPREC112021-16) and the Office for Life Science Research Ethics and Safety, The University of Tokyo (22-99).

### Design

**Fig. 1:**
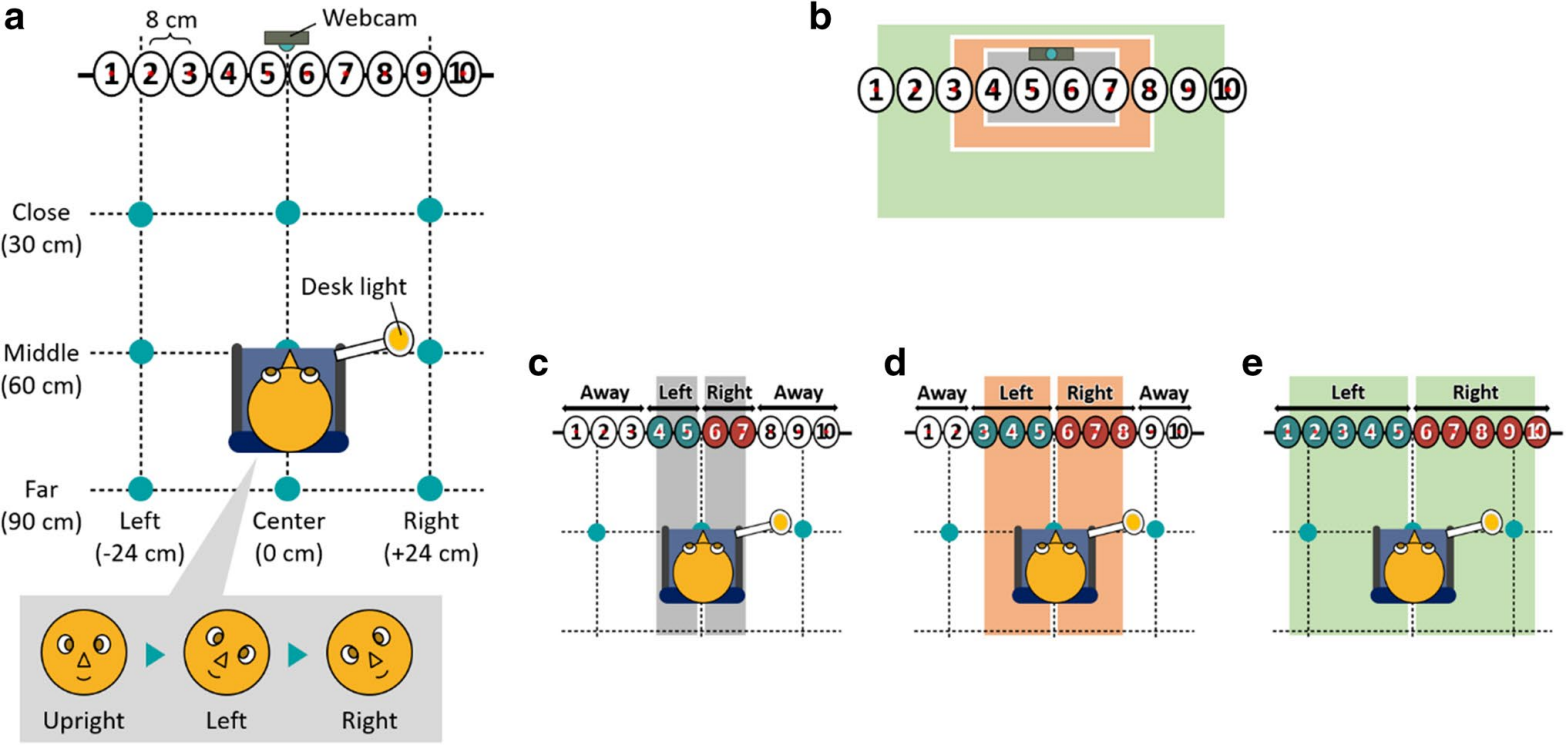
A schematic view of the experimental setting. *Notes*. **a** Overall apparatus. Ten numbered discs were put in a horizontal line on the wall and a webcam was positioned on top of the numbered discs between 5 and 6. Participants were asked to look at the designated number while changing face rotation and the position of the chair to manipulate noise factors frequently observed in infant studies. The position of the desk light was also manipulated. **b** Summary of different assumptions regarding the monitor size and corresponding ground-truth gaze classification: **c** 13” (approximately 29 cm in width); **d** 20” (approximately 44 cm in width); **e** 35” (approximately 77 cm in width). Three categories of looking were considered. When the monitor size of 13” was assumed, looking at the numbered discs 4–5 were considered as looking “Left”, 6–7 as “Right”, and the others as “Away”.

In the experiment, we manipulated four types of noise factors: participants’ left-right offset, distance to the webcam, facial rotation (roll), and the direction of the lighting source. The lighting source was manipulated between subjects whereas the other noise factors were manipulated within subjects. Overall, a single participant engaged in 27 different conditions (3 left-right offsets x 3 distances to the webcam x 3 face rotations x 1 lighting source). Each condition consisted of 20 trials. In each trial, participants were asked to look at designated numbered discs, ranging from 1 to 10, which were positioned in a horizontal line on the wall in front of them. Within each condition, each number was repeated twice. This manipulation was implemented to account for different screen sizes participants might use when participating from their home. Thus, 540 trials in total per participant were recorded, taking approximately 30–40 mins. A schematic view of the experimental settings is shown in Fig. 1.

### Apparatus and materials

For the monitor size manipulation, the numbered discs from 1 to 10 were arranged horizontally on the wall so that the distance between the centers of adjacent discs was 8 cm each (Fig. 1a). Each numbered disc was 8 cm in width and 10 cm in height. The centers of the discs were 113 cm in height from the floor. On top of the numbered discs between 5 and 6, we positioned a webcam (C615, Logitech). As we explain below, participants were asked to look at the numbered disc corresponding to an auditory stimulus (e.g., “one”). This experimental setting, instead of presenting visual stimuli on the monitor, allowed us to conduct a posteriori assessment of the gaze coding algorithm while assuming different monitor widths (Fig. 1b). For instance, if the participants’ monitor size is 13” (approximately 29 cm in width), the numbers 4–5 and 6–7 can be regarded as a gaze to the “left” and “right” side, respectively, and the other numbers can be assigned as “away” from the screen (Fig. 1c, see other assumed situations for Fig. 1d, e).

For the experimental noise manipulation, participants’ positions relative to the camera, face rotations, and lighting source were manipulated. Participants’ left-right offset and distance to the camera were determined based on a previous similar study (Zhang et al., 2019). For left-right offset, three conditions were made: Left (– 24 cm), Center (0 cm), and Right (+ 24 cm). Distance to the camera also had three conditions: Close (30 cm), Middle (60 cm), and Far (90 cm). Thus, there were nine different positions in total. To simplify the experimental procedure, we positioned a chair so that its front legs were aligned with those positional conditions. The distance between the participants’ faces and the webcam was therefore not necessarily identical to the distance specified above. Face rotation had three conditions: Upright, Left, and Right. To manipulate the lighting source, we attached a desk light (800-LED039, SANWA SUPPLY) to the armrest of the chair for the two conditions Left and Right or put it on top of the webcam for the condition Front. During the experiment, the desk light illuminated the participants’ faces and the room light was turned off.

For the audio stimuli, we made 1-sec beep sounds following a voice saying a number between 1 and 10 in either English or Japanese. A specific frequency was assigned to a number so that the frequency of the beep sound got higher as the number uttered increased (e.g., 392 Hz for 5, 440 Hz for 6).

### Procedure

Each participant individually performed the task in an experimental room at Trinity College Dublin (Ireland) or The University of Tokyo (Japan). Participants were seated on a chair oriented towards the numbered discs and were told to look at the numbered discs corresponding to the audio stimulus until the end of the beep sound succeeding each number (e.g., “one...(*beep*), two... (*beep*), ...”). They were instructed to look at the numbers as they naturally would, so they sometimes moved their heads but other times only eyes without head movement. The numbers were played in randomized order.

As to the manipulation of participants’ positions described above, all participants went through nine different chair positions following the same sequence, but with different starting positions. The first position was always Center in terms of left-right offset while the distance to the webcam was counter-balanced. As to ordering, for instance, those who started with the Center-Close position moved to Right-Close, Left-Middle, Center-Middle, Right-Middle, Left-Far, Center-Far, Right-Far, and Left-Close. For the manipulation of face rotation, at each chair position, participants were instructed not to tilt their head (i.e., keep the upright position in terms of roll axis) for the first 20 trials (ten numbers in a random order multiplies two times), then were asked to roll their head to the left-hand side and keep the position for the subsequent 20 trials, and were asked roll their head to the right-hand side for the final 20 trials. The degree to which they tilted their head to the left or right depended on participants' subjective decision, so it varied across participants. Note that they were only asked to maintain their head position in terms of roll, so they could move their head in terms of yaw (and pitch) freely. Each of these within-subject factors was presented twice. For each of the two repetitions of the same condition, the order in which the number sequence was played was identical.

The last manipulation, lighting source, was manipulated between-subjects. Twenty participants each were randomly assigned to lighting from Left, Right, or Front, which was achieved by attaching the desk light to the left or right armrest of the chair or positioning it centrally above the webcam. While participants performed the experimental trials, the overhead room lighting was turned off and the experimenter left the room.

**Fig. 2:**
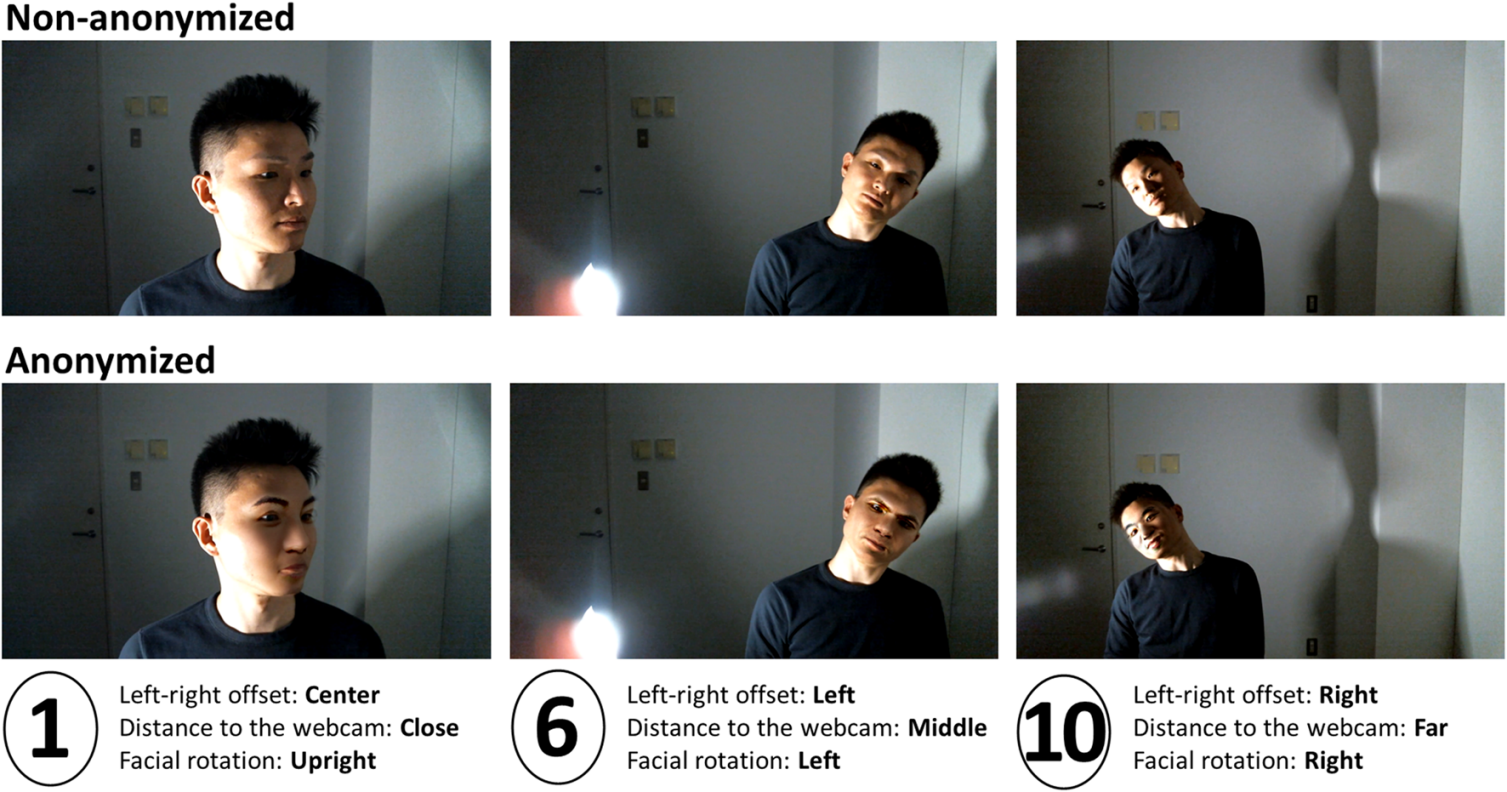
Examples of webcam recordings. *Notes*. Out of the four factors taken into account, the left-right offset, distance to the webcam, and facial rotation (roll) were within-participant factors whereas the lighting source was a between-participant factor. In these examples, the lighting was always shed from the right-hand side. The *numbers* below pictures indicate numbered discs that the participant was looking at

The experimental session was recorded via a webcam (Fig. 2) and took approximately 30–40 min to complete.

### Data analysis

#### Pre-processing

We first cropped video recordings to trial length, defined as the length of the beep sound, which resulted in 540 video files per participant (1 s per file; mean number of frames = 27.8, SD = 6.2, range = 17–33). The number of frames (i.e., frames per second) sometimes decreased due to insufficient amount of lighting^1^. The videos had a spatial resolution of 1920 x 1080. To make sure that participants were properly engaged in the experiment, we manually assessed whether the participants’ looking direction (left, right, away) was the same during the first and second sequences in the same condition in randomly extracted 5% of the videos (14 out of 270 pairs per participant). We obtained a high agreement of 99.5% (only two pairs were considered different in terms of participants’ looking direction and the other two pairs were unable to be assessed due to shadow shed on the participant's face). We can therefore assume that participants were looking at the designated numbers as instructed.

#### Facial anonymization

To create anonymized versions of participants’ facial videos, we ran Deep Natural Anonymization (BrighterAI, n.d.) for all the cropped videos. This detects faces and generates synthetic replacements that reflect original attributes. Thus, we had two video datasets: one with and one without facial anonymization.

#### Automated classification algorithms

Webcam-based automated gaze coding algorithms such as OpenFace (Baltrušaitis et al., 2018), RT-GENE (Fischer et al., 2018), and WebGazer (Papoutsaki et al., 2016) have been actively proposed in the field of computer vision. However, these tools are usually developed based on high-quality videos of still adult faces, and they are often not applicable to online infant studies that come with their own set of noise factors (Erel et al., 2023). To the best of our knowledge, there are at least three automated gaze coding algorithms dedicated to infant looking-time studies: iCatcher+ (Erel et al., 2023), OWLET (Werchan et al., 2022), and an Amazon Rekognition-based model (Chouinard et al., 2019). We chose iCatcher+ as the primary state-of-the-art tool that should be assessed on how noise factors affect its performance because iCatcher+ has been continuously improved, was trained on more than 600 varied video datasets, and requires minimal knowledge of Python, making it attractive both because of its state of the art and its implementability. Erel et al. (2022) reported that OpenFace achieved 51% of correct responses on a tripartite left-right-away categorization, while iCatcher achieved more than 90% of correct responses on the same dataset. This suggests iCatcher+, an improved version of iCatcher, to be the best choice for our current purposes. To confirm the generalizability of the findings from iCatcher+, OWLET analyses were also performed on our data set as a secondary selection, with some technical difficulties described below.

#### *iCatcher+*

On the video datasets, we ran a state-of-the-art classification algorithm, iCatcher+ v0.2.0 (Erel et al., 2023), rooted in computer-vision methods and developed especially for detecting gaze from infant online experiments. iCatcher+ has been developed based on iCatcher (Erel et al., 2022), an automated gaze classifier specifically designed for research with infants and young children. iCatcher+ was reported to achieve accurate and robust gaze classification by being trained on substantially varied datasets in terms of experimental settings (online, in-lab, and outside of the lab), research topics (intuitive physics, language comprehension), infant age and ethnicity. iCatcher+ consists of three components: the face detector, face classifier, and gaze classifier. The face detector extracts areas that possibly include faces using OpenCV (Bradski, 2000), which was not specifically tuned toward infant face detection. Candidate areas are then fed into the face classifier. This determines whether the area is an infant's or adult’s face and selects which of the candidate areas is most likely to belong to the participant. The gaze classifier then predicts gaze direction (i.e., Left, Right, and Away). It produces this result for the temporal middle frame within the moving window of five consecutive frames. If no face is found, the model returns a label of Invalid. As to the predicted gaze directions, Left and Right are defined as frames in which the infant is looking to the left or right side of the screen, respectively. Away is defined as frames in which the infant is looking somewhere else than the screen, such as turning around to look at the caregiver or looking at her feet (see Erel et al., 2023 for more details). For the present study, we did not use the face classifier from iCatcher+ and performed only the face detector and gaze classifier because our dataset consisted of adult webcam videos with only one human face^2^.

#### *OWLET*

OWLET, an online webcam-linked eye-tracker (Werchan et al., 2022), is an open-source methodology for automatically estimating infants’ gaze coordinates on the monitor based on smartphone and webcam recordings. High correlation coefficients of over 0.95 were reported between OWLET-human annotations in terms of overall looking time, maximum looking duration, and the number of gaze shifts. OWLET consists of three components: infants’ face/eye/pupil detector, gaze direction estimator, and estimator of point-to-gaze on the screen. The first component extracts infants’ face/gaze/pupil frame-by-frame using OpenCV (Bradski, 2000) and Dlib Machine Learning Toolkit (King, 2009). If more than one face is extracted, the lower face is processed thereafter. The isolated pupils are fed into the gaze direction estimator, which calculates gaze direction while taking infants’ eye and head position into account. Finally, the gaze direction is mapped to precise screen (*x*, *y*) coordinates using a simple polynomial transfer function. A bipartite looking-away categories were also produced. A six-frame moving average filter is applied in this phase for smoothing and the model produces the gaze coordinates at a temporal resolution of 30 Hz. OWLET is designed to perform optimally under the usage of a four-point calibration before experiments.

Since OWLET was published after we created the dataset, we did not record each participant's calibration. We used OWLET with the commits on March 2023 and default calibration settings were applied. We also removed from the source code the required temporal resolution of 30 Hz as most of our videos were below this threshold. To produce gaze categories equivalent to iCatcher+, we first classified output frames without (*x*, *y*) coordinates as No Face. We next classified frames for which (*x*, *y*) coordinates were estimated but the Away category was adopted on the looking-away column as Away. We then classified the remaining frames into Left or Right depending on their estimated coordinates. Since OWLET always assumes the monitor’s spatial resolution of 960 x 540 (16:9), we assigned those frames' gaze categories based on the median in terms of *x*-coordinate (960 / 2 = 480).

#### Missing data

iCatcher+ succeeded in processing all the videos, whereas OWLET produced error messages for a total of 143 videos (0.22%): 42 videos for non-anonymized (0.13%, 0–5 videos per participant) and 101 videos for anonymized (0.31%, 0–16 videos per participant). As far as we understood, the errors were caused either because the process stopped when eyes were not found in the second frame despite successful detection in the first frame (111 videos) or because the coordinates of different eye landmarks were determined to be identical for some reason (32 videos). We removed those unprocessable files from the analyses.

#### Regression analyses

We performed generalized linear mixed models (binomial regression) independently for each dataset (i.e., with or without anonymization) to assess the effects of environmental and behavioral factors on two different dependent variables: (1) whether faces were detected; (2) how accurately gaze directions were estimated. For (1), given that participants were seated in front of the camera throughout the experiment, we can assume that all frames analyzed contained faces. If the face was successfully detected by the algorithm, the value 1 was given whereas if no face was detected, the value 0 was given. For each video file, the proportion of frames in which the face was detected out of the total number of frames was regarded as the dependent variable. For (2), we adopted three different assumptions of monitor size and corresponding ground-truth gaze classifications (see Fig. 1c–e): Small (approximately 30 cm in width), Medium (approximately 46 cm), and Large (approximately 78 cm). As mentioned above, if the participants’ monitor size is assumed Small, the numbered discs 4-5 are classified as “Left”, 6-7 as “Right”, and the others as “Away”. Given that we had estimated participants’ engagement in the experiment in the preprocessing stage, we defined the true gaze direction as the direction the participant was instructed to look towards (i.e., the number they heard), and corresponding gaze classifications were coded for each monitor size assumption. We first excluded frames with the failure of face detection from each video. For each frame, if the estimated gaze direction (i.e., Left, Right, Away) matched the true direction (see Fig. 1b), the value 1 was given, whereas if they were different, the value 0 was given. We then calculated the proportion of frames with the correct estimation out of the total number of frames with face detection. For instance, if a video had 20 frames with 2 of them as No Face, 10 as Left, 3 as Right, and 5 as Away, and its corresponding true direction was Left, the proportion of correct estimation was 55.6% (= 10 / (10+3+5)). We also decided on a single gaze coding estimation per video on a majority-vote basis to calculate confusion matrices (in this example, Left). Files in which no face was detected for all the frames were removed from this analysis. The regression analysis was performed using *lme4* version 1.1.31 (Bates et al., 2015) on *R* version 4.2.2 (R Core Team, 2022).

The fixed effects included left-right offset (Left, Center, Right), distance to the camera (Close, Middle, Far), facial rotation (Left Upright, Right), and the direction of the lighting source (Left, Front, Right). The country (Ireland, Japan) was also included as a covariate. The reference level was specified as the ideal combination of the conditions (left-right offset: Center, distance: Close, facial rotation: Upright, lighting source: Front) and the country (Ireland) for convenience. We included the random intercept of participants for each model. Because our primary purpose here was to assess the effects of noise factors on facial detection and gaze classification, not those of cultural differences, we report cumulative results across the two countries for the sake of legibility except when a significant effect of the country is present (results by country are shown in Supplementary Materials).

## Results

### Facial detection

We first assessed to what extent the participants’ faces were detected properly. Overall, iCatcher+ successfully detected faces for almost all the frames, achieving 99.9% for both Non-anonymized and Anonymized datasets. It failed to detect faces in only one video file for each dataset (1 frame in one video for Non-anonymized dataset and the entire 10 frames in another video for Anonymized dataset). On the other hand, OWLET detected faces in 46.4% of all frames for Non-anonymized dataset and 62.9% for Anonymized dataset, respectively. Since the manipulated environmental and behavioral factors affected only face detection in OWLET and not in iCatcher+, we focused on examining the extent to which each factor affected face detection in OWLET only.

For the Non-anonymized dataset, the proportion of face detection significantly dropped when participants’ faces were not positioned at the center in terms of left-right offset (Left: *b* = – 0.15, *SE* = 0.01,* p* < .001; Right: *b* = – 0.10, *SE* = 0.01,* p* < .001), participants’ faces were not upright (Left: *b* = – 0.18, *SE* = 0.01,* p* < .001; Right: *b* = – 0.65, *SE* = 0.01,* p* < .001), and the lighting source was not located in front of the participants (Left: *b* = – 2.49, *SE* = 0.34,* p* < .001; Right: *b* = – 2.69, *SE* = 0.35,* p* < .001). Face detection performance also significantly dropped for participants in Japan (*b* = – 0.70, *SE* = 0.29,* p* < .014). On the other hand, face detection was significantly greater when the distance to the camera was Middle (*b* = 0.17, *SE* = 0.01,* p* < .001) and Far (*b* = 0.11, *SE* = 0.01,* p* < .001). We did not include interaction effects in our model, but in general, faces were properly detected as long as the lighting source was located in front of the participants (Fig. S1). If this condition was met, even the least ideal condition (left-right offset: Left, distance: Close, facial rotation: Right, lighting source: Front) demonstrated a moderate facial detection ability above chance (Ireland: 79.1%, 95% CI [68.6, 86.7]; Japan: 65.1%, 95% CI [52.0, 76.4]). The significant effect of country may need a careful interpretation because, although we attempted to make the experimental settings as similar as possible, some uncontrolled factors such as the physical properties of the experimental room and participants’ race may have caused confounds.

A comparable result was obtained when OWLET was performed for the Anonymized dataset, except for a significant positive effect of face rotation to the Left (*b* = 0.10, *SE* = 0.01,* p* < .001; see Table S2) on face detection. As in the Non-anonymized data, if the lighting source was located in front, faces were still detected well in the worst condition (left-right offset: Left, distance: Close, facial rotation: Right, lighting source: Front; Ireland: predicted proportion = 79.0%, 95% CI [70.7, 85.4]; Japan: 62.3%, 95% CI [51.4, 72.0]; see Fig. S1).

## Gaze direction classification

We then assessed how accurately gaze directions were estimated for different conditions while varying the assumptions of monitor size. For iCatcher+, one video in the Anonymized dataset was removed from the analysis because it failed to detect faces for all the frames. For OWLET, 13,254 videos (40.1%) in the Non-anonymized and 4874 videos (15.1%) in the Anonymized datasets were also removed due to face detection failure. The overall proportion of correct predictions by iCatcher+ was above chance (33.3%) but below 60% regardless of whether the assumed monitor size was Small (Non-anonymized: 44.4%; Anonymized: 40.6%), Medium (Non-anonymized: 41.9%; Anonymized: 39.7%), or Large (Non-anonymized: 46.5%; Anonymized: 46.3%). The overall proportion of correct predictions by OWLET was generally lower than iCatcher+ when the assumed monitor size was Small (Non-anonymized: 26.0%; Anonymized: 25.1%) or Medium (Non-anonymized: 36.9%; Anonymized: 35.7%), but better when the assumed monitor size was Large (Non-anonymized: 57.8%; Anonymized: 56.7%).

### iCatcher+

We first report the prediction performance of iCatcher+ for the Non-anonymized data. When assuming Small monitor size (Fig. 1c), we found significant negative effects of the distance to the camera (Middle: *b* = – 0.18, *SE* = 0.01,* p* < .001; Far: *b* = – 0.12, *SE* = 0.01, *p* < .001), facial rotation (Left: *b* = – 0.16, *SE* = 0.01,* p* < .001; Right: *b* = – 0.07, *SE* = 0.01, *p* < .001), and the lighting source (Left: *b* = – 0.53, *SE* = 0.07,* p* < .001; Right: *b* = – 0.43, *SE* = 0.07, *p* < .001). We found a significant positive effect when the position was offset toward the Left (*b* = 0.07, *SE* = 0.01,* p* < .001), whereas we found a non-significant effect when it was offset toward the Right (see Table S3 for details). The performance was significantly higher for Japanese participants (*b* = 0.22, *SE* = 0.06,* p* < .001). The predicted proportion of correct estimation in the condition with the highest performance (left-right offset: Left, distance: Close, facial rotation: Upright, lighting source: Front) was 50.8% (Ireland: 48.1%, 95% CI [45.3, 50.9]; Japan: 53.4%, 95% CI [50.6, 56.2]; see Fig. S2). In this condition, overall classification accuracy was 58.0% (Left: 76.2%, Right: 37.5%, Away: 58.8%; F1 = 53.6%). The worst condition (left-right offset: Right, distance: Middle, facial rotation: Left, lighting source: Left) showed a predictability of 28.8% (Ireland: 26.6%, 95% CI [24.4, 28.8]; Japan: 31.0%, 95% CI [28.6, 33.5]). The overall classification accuracy for this condition was 31.8% (Left: 21.2%, Right: 71.2%, Away: 22.1%; F1 = 30.1%).

**Fig. 3:**
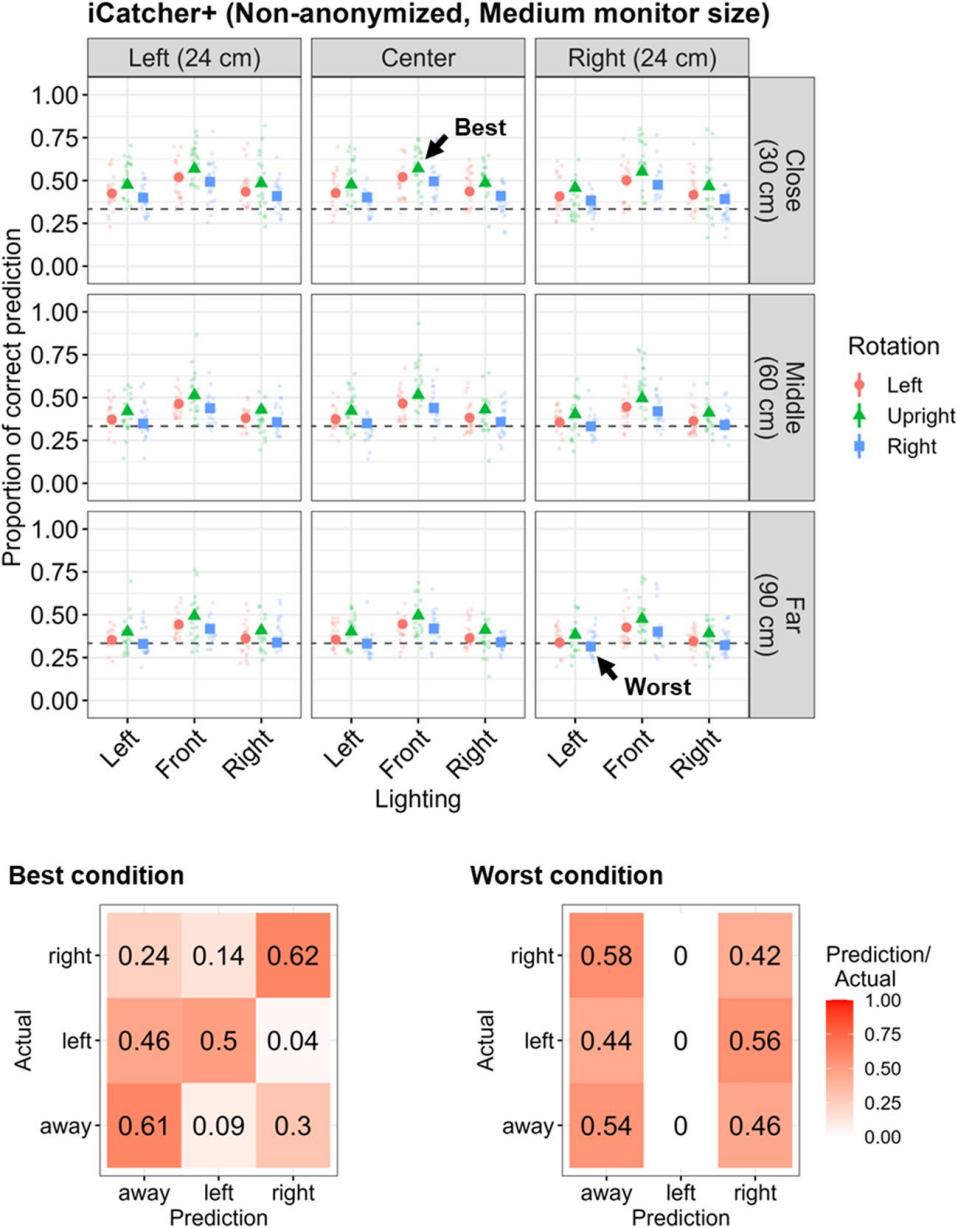
Predictability of gaze direction by iCatcher+ on the Non-anonymized dataset assuming Medium monitor size. *Notes*. The top figure shows the proportion of correct predictions across different noise conditions. The *colored points* and their range represent the predicted values and 95% confidence intervals, respectively. The *dashed horizontal line* indicates the chance level. The *translucent jittered points* represent the mean proportion of correct prediction for each participant. The distance to the webcam, facial rotation, and lighting sources negatively affected the predictability of gaze coding by iCatcher+. The left-right offset did not have a robust negative effect, and the regression coefficient was relatively small even if it was significant. The bottom panels represent the confusion matrix for the best (*left*) and worst (*right*) conditions. The overall classification accuracy in the best condition was 58.0%, whereas that in the worst condition was 49.3% and no videos were predicted as “left”

When assuming Medium monitor size (Fig. 1d), the distance to the camera (Middle: *b* = – 0.22, *SE* = 0.01,* p* < .001; Far: *b* = – 0.30, *SE* = 0.01,* p* < .001), facial rotation (Left: *b* = – 0.20, *SE* = 0.01,* p* < .001; Right: *b* = – 0.30, *SE* = 0.01,* p* < .001), and lighting source (Left: *b* = – 0.38, *SE* = 0.05,* p* < .001; Right: *b* = – 0.34, *SE* = 0.05,* p* < .001) negatively affected the predictability of gaze directions by iCatcher+. The offset toward the Right also had a negative effect (*b* = – 0.08, *SE* = 0.01,* p* < .001), whereas the offset toward the Left did not have a significant effect (see Table S3). The country did not significantly affect the performance. In the best condition (left-right offset: Center, distance: Close, facial rotation: Upright, lighting source: Front), the predicted proportion of correct estimation was 56.9% (Ireland: 57.0%, 95% CI [55.1, 58.9]; Japan: 56.8%, 95% CI [54.8, 58.7]), whereas that in the worst condition (left-right offset: Right, distance: Far, facial rotation: Right, lighting source: Left) was 31.3% (Ireland: 31.4%, 95% CI [29.8, 33.1]; Japan: 31.2%, 95% CI [29.5, 32.9]; Fig. 3). The overall classification accuracy was 58.0% (Left: 50.0%, Right: 61.7%, Away: 61.2%; F1 = 58.0%) in the best condition and 34.5% (Left: 0.0%, Right: 42.5%, Away: 54.4%; F1 = 40.0%) in the worst condition.

When assuming Large monitor size (Fig. 1e), all the noise factors showed significant negative effects on the proportion of correct gaze classification, except for the non-significant effects of lighting source (see Table S2). The predicted proportion of correct classification in the best condition (left-right offset: Center, distance: Close, facial rotation: Upright, lighting source: Left) was 62.4% (Ireland: 69.0%, 95% CI [63.1, 74.4]; Japan: 55.8%, 95% CI [49.2, 62.2]), whereas that in the worst condition (left-right offset: Right, distance: Far, facial rotation: Right, lighting source: Front) was 27.3% (Ireland: 32.9%, 95% CI [27.4, 38.9]; Japan: 21.7%, 95% CI [17.6, 26.5]; Fig. S2). In the best condition, the overall classification accuracy showed 74.2% (Left: 64.0%, Right: 84.5%; F1 = 78.2%). That in the worst condition was 31.8% (Left: 22.5%, Right: 41.0%; F1 = 41.7%).

We also performed iCatcher+ classification for the Anonymized data. The results between the Non-anonymized and Anonymized datasets were largely comparable. For details, see Supplementary Materials (Table S4 and Fig. S3). Compared to the Non-anonymized data, the predictability for the Anonymized data was generally slightly lower for both the best (difference: *M* = 4.9%, *SD* = 5.8, range = – 1.5–10.0) but equivalent for the worst (*M* = – 0.6%, *SD* = 1.6, range = – 2.3–0.9) conditions. The overall classification accuracy was also lower for the best (difference: *M* = 8.8%, *SD* = 7.9, range = 1.2–17.0) and worst (*M* = 1.0%, *SD* = 1.2, range = – 0.4–1.8) conditions accordingly.

### OWLET

We next report the prediction performance of OWLET for the Non-anonymized data. When assuming Small monitor size (Fig. 1c), we found significant negative effects of the distance to the camera (Middle: *b* = – 0.54, *SE* = 0.01,* p* < .001; Far: *b* = – 0.75, *SE* = 0.01, *p* < .001) and the lighting source (Left: *b* = – 0.40, *SE* = 0.08,* p* < .001; Right: *b* = – 0.45, *SE* = 0.08, *p* < .001) as seen in the results of iCatcher+. Different from iCatcher+, facial rotation had significant positive effects (Left: *b* = 0.03, *SE* = 0.01,* p* = .001; Right: *b* = – 0.20, *SE* = 0.01, *p* < .001). The left-right offset did not have consistent negative effects and the country did not affect the performance of OWLET (see Table S5 for details). The predicted proportion of correct estimation in the condition with the highest performance (left-right offset: Center, distance: Close, facial rotation: Right, lighting source: Front) was 41.1% (Ireland: 40.4%, 95% CI [37.4, 43.5]; Japan: 41.7, 95% CI [38.6, 44.8]; see Fig. S4). In this condition, overall classification accuracy was 41.8% (Left: 13.9%, Right: 47.1%, Away: 49.8%; F1 = 35.1%). The worst condition (left-right offset: Left, distance: Far, facial rotation: Upright, lighting source: Right) showed a predictability of 14.2% (Ireland: 13.9%, 95% CI [12.4, 15.5]; Japan: 14.5%, 95% CI [13.0, 16.2]). The overall classification accuracy for this condition was 18.6% (Left: 97.3%, Right: 0.0%, Away: 0.0%; F1 = 31.9%).

**Fig. 4:**
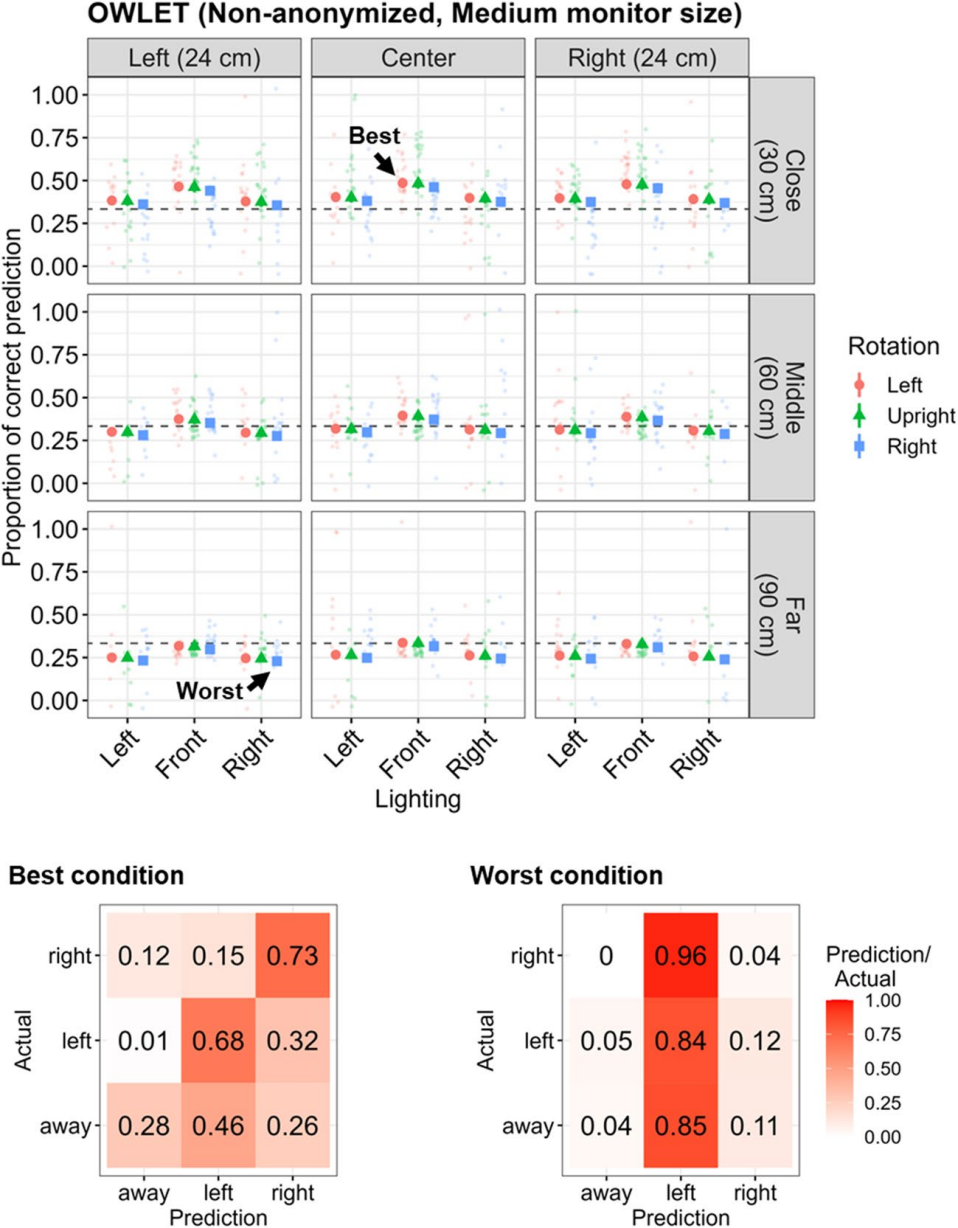
Predictability of gaze direction by OWLET on the Non-anonymized dataset assuming Medium monitor size. *Notes*. The specification is the same as in Fig. 3. The distance to the webcam, left-right offset, and lighting sources negatively affected the predictability of gaze coding by OWLET. Facial rotation did not have a robust negative effect, and the regression coefficient was relatively small even if it was significant. The overall classification accuracy in the best condition was 53.7%, whereas that in the worst condition was 28.2%

When assuming Medium monitor size (Fig. 1d), the distance to the camera (Middle: *b* = – 0.37, *SE* = 0.01,* p* < .001; Far: *b* = – 0.62, *SE* = 0.01,* p* < .001), left-right offset (Left: *b* = – 0.08, *SE* = 0.01,* p* < .001; Right: *b* = – 0.03, *SE* = 0.01,* p* < .001), and lighting source (Left: *b* = – 0.33, *SE* = 0.08,* p* < .001; Right: *b* = – 0.35, *SE* = 0.08,* p* < .001) negatively affected the predictability of gaze directions by OWLET. Facial rotation toward the Right also had a negative effect (*b* = – 0.08, *SE* = 0.01,* p* < .001), whereas facial rotation toward the Left did not have a significant effect (see Table S5). The country did not significantly affect the performance. In the best condition (left-right offset: Center, distance: Close, facial rotation: Left, lighting source: Front), the predicted proportion of correct estimation was 49.4% (Ireland: 48.5%, 95% CI [45.5, 51.6]; Japan: 50.3%, 95% CI [47.3, 53.4]), whereas that in the worst condition (left-right offset: Left, distance: Far, facial rotation: Right, lighting source: Right) was 23.6% (Ireland: 22.9%, 95% CI [20.8, 25.2]; Japan: 24.2%, 95% CI [22.0, 26.5]; Fig. 4). The overall classification accuracy was 53.7% (Left: 67.5%, Right: 72.9%, Away: 28.3%; F1 = 52.7%) in the best condition and 28.2% (Left: 83.7%, Right: 4.3%, Away: 3.7%; F1 = 18.9%) in the worst condition.

When assuming Large monitor size (Fig. 1e), facial rotation (Left: *b* = – 0.02, *SE* = 0.01,* p* = .01; Right: *b* = – 0.58, *SE* = 0.01,* p* < .001) and the lighting source (Left: *b* = – 0.25, *SE* = 0.10,* p* = .008; Right: *b* = – 0.22, *SE* = 0.10,* p* = .02) showed significant negative effects on the proportion of correct gaze classification. We also found a significant negative effect when the distance to the camera was Far (*b* = – 0.35, *SE* = 0.01, *p* < .001), while we found a significant positive effect when the distance was Middle (*b* = 0.04, *SE* = 0.01, *p* < .001). The offset towards the Left had a significant negative effect (*b* = – 0.07, *SE* = 0.01, *p* < .001), but a non-significant effect of the offset towards the Right (see Table S5). The predicted proportion of correct classification in the best condition (left-right offset: Center, distance: Middle, facial rotation: Upright, lighting source: Front) was 62.4% (Ireland: 68.5%, 95% CI [64.1, 70.9]; Japan: 69.4%, 95% CI [66.0, 72.6]), whereas that in the worst condition (left-right offset: Left, distance: Far, facial rotation: Right, lighting source: Left) was 37.4% (Ireland: 36.4%, 95% CI [32.9, 40.1]; Japan: 38.4%, 95% CI [34.7, 42.1]; Fig. S4). In the best condition, the overall classification accuracy showed 68.3% (Left: 100.0%, Right: 36.5%; F1 = 64.7%). That in the worst condition was 51.6% (Left: 93.6%, Right: 9.1%; F1 = 40.9%).

We also performed OWLET classification for the Anonymized data. The results between the Non-anonymized and Anonymized datasets were largely comparable (see Supplementary Table S6 and Fig. S5 for details). Compared to the Non-anonymized data, the predictability for the Anonymized data was generally slightly lower for the best (difference: *M* = 1.7%, *SD* = 1.5, range = 0.0–3.0) and slightly higher for the worst (*M* = – 2.6%, *SD* = 0.4, range = – 2.0 to –2.2) conditions, respectively. The overall classification accuracy was equivalent for both the best (difference: *M* = – 0.7%, *SD* = 1.5, range = – 2.4 to 0.3) and worst (*M* = 0.3%, *SD* = 1.3, range = – 0.9 to 1.6) conditions.

### Summary

**Fig. 5:**
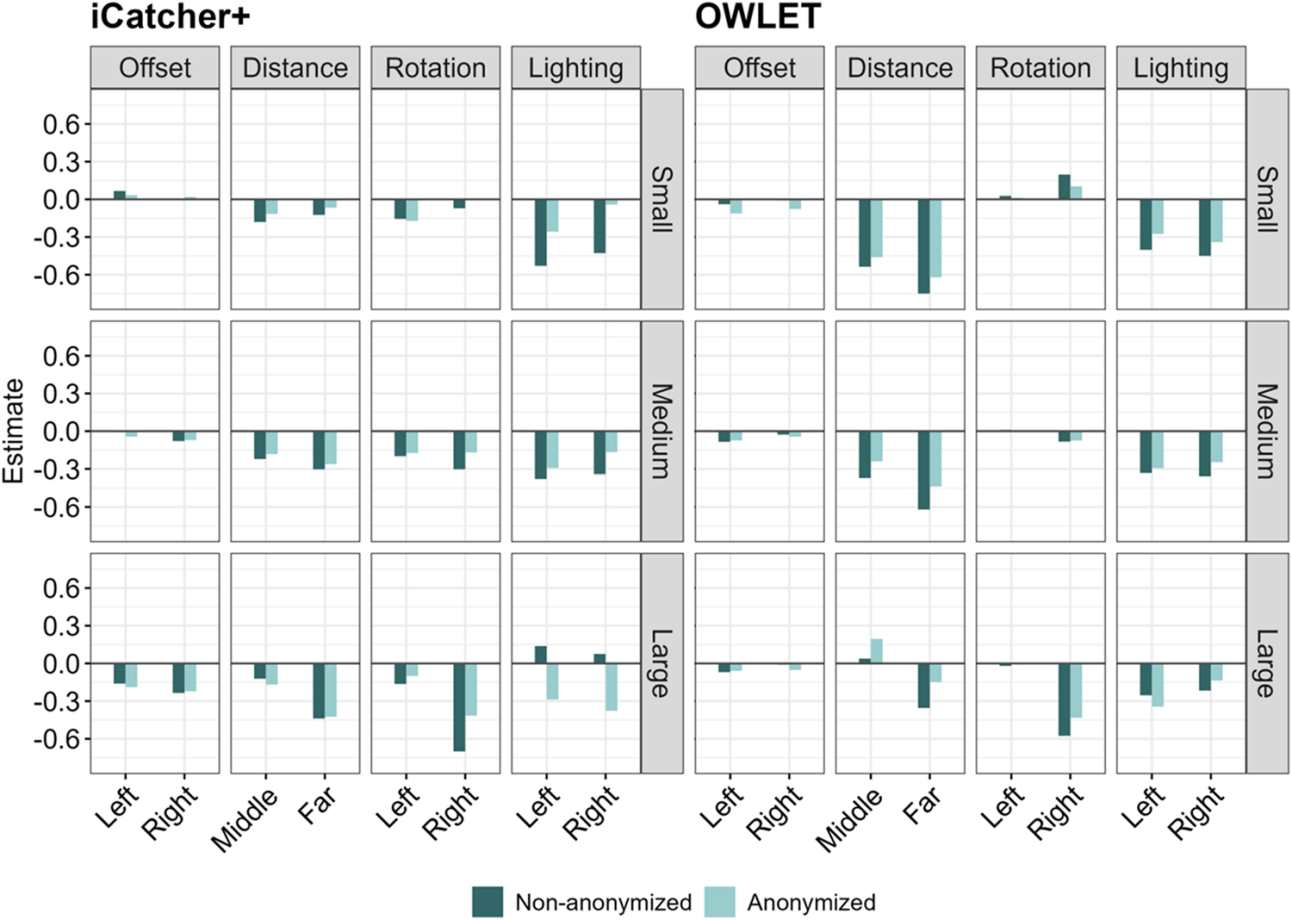
Summary of regression coefficients of noise factors on gaze coding accuracy. *Notes*. Regression coefficients of each noise factor (left-right offset, distance to the camera, facial rotation, and lighting source) across different algorithms and different assumed monitor sizes are visualized. In general, the distance to the webcam and lighting source consistently had negative effects on gaze coding accuracy. The facial rotation also negatively affected the performance of iCatchet+. The left-right offset had little influence on gaze coding accuracy. The results between Non-anonymized and Anonymized datasets were largely compatible

In general, the distance to the webcam and the lighting source consistently affected automated gaze coding by iCatcher+ or OWLET regardless of different monitor size assumptions and whether faces were anonymized or not, albeit with a few exceptions such that the lighting source had less influence on iCatcher+ when the assumed monitor size was Large for the Non-anonymized dataset and the Middle distance had conversely positive effects on this gaze coding by OWLET (Fig. 5). Left-right offset also negatively influenced the gaze coding accuracy for both algorithms, but the magnitude of its impact was relatively small, considering the regression coefficients were constantly small and they were sometimes not significant depending on the assumed monitor size. While facial rotation constantly worsened the gaze coding performance of iCatcher+ regardless of the assumed monitor size and face anonymization, it sometimes showed even positive effects on the performance of OWLET when the assumed monitor size was Small.

Differences in the classification of gaze direction were more pronounced across conditions and the predictability of gaze direction was superior as the assumed monitor size became larger. However, even in the most ideal condition, the overall classification accuracy remained at around 60–70% and asymmetric patterns in accuracy were found between gaze classification of Left or Right (see also Figs. S6 and S7 for how each algorithm predicted the participants’ gaze direction corresponding to each numbered disc). In the worst conditions, this asymmetry was even more pronounced. The gaze prediction performance was slightly lower for the Anonymized dataset than the Non-anonymized dataset when the noise factors were well controlled (i.e., best conditions), suggesting that facial features that are important to predict gaze directions might be somewhat distorted or lost after the facial anonymization. On the other hand, the performance was conversely better for the Anonymized dataset compared with the Non-anonymized dataset when the noise factors were greatly contaminated (i.e., worst conditions), suggesting that some of the lost facial features were somewhat recovered by facial anonymization.

## Discussion

In this study, we created an adult webcam dataset that systematically reproduced four types of noise factors likely present in at-home online gaze experiments with young children and investigated to what extent each factor relatively affected gaze coding accuracy by two state-of-the-art algorithms for infant experiments: iCatcher+ (Erel et al., 2023) and OWLET (Werchan et al., 2022). We primarily focused on two outcomes: Whether faces were successfully detected and whether gaze directions were accurately estimated. Regardless of whether participants’ faces were anonymized or not, their faces were successfully and reliably detected as long as the lighting source was located in front of the participant. Among the four different noise factors that were tested, the distance to the webcam and lighting source consistently decreased gaze coding accuracy, and the facial rotation also negatively affected the performance of iCatchet+ in particular. A practically beneficial finding is that the left-right offset had little influence on gaze coding accuracy.

For extracting participants’ facial features automatically from online data, our results suggest that, at least among the four noise factors considered in this study, the participant mainly needs to be instructed not to locate the lighting source to the left or right so as not to project shadows on their face. This is essential especially when a researcher plans to use OWLET for processing the data, while the lighting source will not matter for the face detection itself by iCatcher+. This discrepancy might be explained by the fact that OWLET uses Dlib Machine Learning Toolkit (King, 2009) in addition to OpenCV (Bradski, 2000) to extract infants’ face/gaze/pupil, while iCatcher+ uses only OpenCV. Future work would need to verify whether other state-of-the-art face detection algorithms can lead to finding faces more successfully in challenging situations. For instance, Chouinard et al. (2019) used Amazon Rekognition (Amazon, n.d.) and Hagihara et al. (2021) used OpenFace (Baltrušaitis et al., 2018), respectively.

The classification accuracy for iCatcher+ was much lower than the previously reported > 80% (Erel et al., 2023) even in the ideal condition in our study (approximately 50–60%). Although our main focus was to assess the relative effects of noise factors and anonymization on existing automated gaze coding algorithms and not to assess the algorithms’ performance itself, it would be worthwhile to consider what caused this discrepancy. One possibility is just a qualitative difference between infants’ and adults’ faces and gaze/face movements. Another possibility is that the experimental settings in this study may have overestimated the degree of noise factors. For instance, although we turned off the room light and a desk light was the only lighting source, real infant conditions might be slightly better for automated gaze coding because the screen itself illuminates their faces to a certain extent. Moreover, the way we operationalized looks away from the screen might have made their categorization more difficult than would happen in the real world and in the data iCather+ was trained on. The primary situations where gaze behavior should be coded as Away in real world settings are when the infant is looking around the room or turning around to look at the caregiver, which would often result in a visual angle distinct from an on-screen look, up to facial occlusion. In our experiment, Away was required to detect a much more subtle effect, namely looks beyond the boundary of a certain screen size. Although we deliberately chose this manipulation in an aim to create a more nuanced video dataset that involved sufficiently difficult discriminations to probe the performance of existing gaze coding algorithms, this might have made results worse than would occur in reality, since anecdotally speaking, infants in real settings would not frequently look beyond the boundaries of the screen. Our setup may thus have worsened the overall performance compared to natural at-home data collection. Nevertheless, we believe our results have practical implications for researchers who plan to do online testing with infants or use iCatcher+ or OWLET, as optimizing the performance and avoiding any decrease in accuracy is crucial for the validity of these methods.

In addition to investigating how much noise factors affect the performance of automated gaze coding, we also applied a state-of-the-art facial anonymization technique (Deep Natural Anonymization; BrighterAI, n.d.) to our adult dataset and assessed how compatible the results with and without facial anonymizations were. The automated facial detection worked equivalently well or even better for the Anonymized dataset compared to the Non-anonymized dataset. The gaze coding accuracy was slightly less accurate for the Anonymized dataset in relatively well-controlled conditions, whereas this tendency flipped in relatively less controlled conditions, that is, the performance was better for the Anonymized dataset than the Non-anonymized dataset. This implies that the facial anonymization technique used in this study might have exaggerated participants' facial features, but at the same time, this might also have led to distorting their gaze and/or facial features and their combinations from the original data (see Fig. 2 for some examples). Yet, given that the predictability of gaze coding for the Anonymized data was just slightly lower for the data with the least noise contamination, this method can be applicable for real webcam videos if noise factors are well controlled. One of the next steps will be to apply the facial anonymization technique to publicly available infants’ webcam videos (e.g., Scott et al., 2017) to extend our findings and confirm that facial anonymization does not destroy the gaze features of the original data even for infants. If this is the case, then developmental scientists will have obtained a new option to enhance open science by sharing infants' video datasets while their faces are kept unidentifiable.

The dataset created in this study paves the way for various future studies. For example, other noise factors listed in previous studies (e.g., Bánki et al., 2022) can be taken into account by post hoc editing of the dataset. The spatial resolution, overall brightness, and facial occlusion can be varied by video manipulation and then assessed as in this study. Another ambitious possibility would be to revise or even develop a more sophisticated automated gaze coding platform. In many cases, iCatcher+ and OWLET showed asymmetric patterns for prediction accuracy. In some cases, Left was predicted more correctly than Right or Left was more likely to be predicted over different numbered discs, and vice versa (Figs. S6 and S7). Such asymmetricity could be modified by feeding our dataset to the model and retraining it in combination with other datasets. The implementation of the calibration phase, as in OWLET, may prevent such asymmetric prediction by defining the center and edge of the monitor. Incorporating the information about monitor size may also be useful to discriminate gaze direction more accurately. Bánki et al. (2022) reported that the majority of the participants used a monitor with a screen size of 15” or below when performing online testing, suggesting the need to process nuanced gaze behavior. Since 78% of our dataset (*n* = 47) is publicly available, researchers can re-use them to tackle different practical issues in webcam-based experiments.

To the best of our knowledge, this is the first study which created webcam video datasets that systematically introduced noise factors frequently observed in infant studies and assessed how they impact automated gaze coding. Recent studies have also tackled relevant topics, suggesting that webcam-based automated gaze coding has become even more important. For instance, Valtakari et al. (2023) assessed the accuracy of existing gaze coding algorithms (e.g., OpenFace) for infant testing and showed its potential utility. However, they did not provide raw video data recorded by a webcam, limiting the possibility of secondary analyses using different algorithms. Their findings also seem difficult to be directly applied to online testing from home environments because, in their set-ups, two webcams were located near the target objects positioned side-by-side in front of infants. In contrast, our video dataset and findings will guide the design of better instructions for participants during online experiments for infants. Guided by our findings, investing time and effort into giving more targeted instructions for optimizing the data quality will lead to more efficient data processing. Moreover, training algorithms using the dataset, or designing new ones robust to the variations, will allow researchers to improve robustness and allow more developmental psychologists to leverage online testing more efficiently.

### Supplementary Information

Below is the link to the electronic supplementary material.

Supplementary file1 (PDF 4.14 MB)

## Data Availability

The videos for which participants agreed to public availability (*n* = 47) are uploaded to https://doi.org/10.17605/OSF.IO/48ZVH.

## Footnotes

1 Video framerates on webcams generally vary depending on lighting exposure, as the shutter stays open longer to let more light in to achieve sufficient lighting exposure, leading to lower framerates.

2 The latest version of iCatcher+ includes the lowest-face selector by default as in OWLET, instead of the face classifier that was originally used (for more details, see https://github.com/icatcherplus/icatcherplus.github.io).

## Code availability

The codes for data preprocessing and analysis are available at https://github.com/hagi-hara/adult-gaze-coding.
