# Supplementary material for: Exploration of factors affecting webcam-based automated gaze coding
Source: Behav Res Methods. 2024 May 1;56(7):7374–90. doi: 10.3758/s13428-024-02424-1 (PMC11362184; doi:10.3758/s13428-024-02424-1)
Supplement: Supplementary file 1 — Supplementary file1 (PDF 4.14 MB) [file 13428_2024_2424_MOESM1_ESM.pdf]

Supplementary Materials

Table S1

A pilot classification of noise factors frequently observed in infant online experiments

Factors	Description	%Videos
Environmental Factors		
Lighting	Too bright or dark lighting to detect the child’s face, a biased lighting source that makes a shadow on the child’s face.	36 %
Camera	Too low spatial resolution to detect the child’s face, relocation of a webcam.	34 %
Another person	An unrelated third party caught on a webcam.	18 %
Infants’ Behavioral Factors		
Occlusion	The child’s face is partially or entirely occluded (not by the caregiver’s body).	92 %
Head movement	The child’s head drastically moves so that his/her face is difficult to be detected.	85 %
Position	The child’s face is not positioned properly, e.g., not being at the center of a webcam's view field.	79 %
Face rolling	The child’s face is not upright.	34 %
Eye closing	The child closes his/her eyes while his/her face is towards the monitor.	10 %
Caregivers’ Behavioral Factors		
Position	The caregiver’s face is too close to the child’s face.	89 %
Body movement	The caregiver drastically moves so that the child’s face is difficult to be detected.	21 %
Occlusion	The child’s face is partially or entirely occluded by the caregiver’s body.	8 %

Notes. Based on Hagihara et al. (2022), which exploratorily classified noise factors that can worsen webcam-based automated gaze coding. The video dataset used in this pilot classification (*n* = 61; https://osf.io/mbcu2) consists of two existing infant online experiments using Lookit platform (Scott et al., 2017; Scott & Schulz, 2017): “Oneshot study” (19 publicly available videos of infants from 11 to 17 months of age) and “NovelVerbs study” (42 publicly available videos of infants from 24 to 36 months of age).

Table S2
The proportion of faces detected by OWLET

	Non-anonymized			Anonymized		
Term	Estimate	SE	*p*-value	Estimate	SE	*p*-value
Intercept	**2.12**	**0.28**	**< .001**	**1.60**	**0.23**	**< .001**
Left-right offset						
Left	**-0.15**	**0.01**	**< .001**	**-0.18**	**0.01**	**< .001**
Right	**-0.10**	**0.01**	**< .001**	**-0.05**	**0.01**	**< .001**
Distance						
Middle	**0.17**	**0.01**	**< .001**	**0.20**	**0.01**	**< .001**
Far	**0.11**	**0.01**	**< .001**	**0.25**	**0.01**	**< .001**
Facial rotation						
Left	**-0.18**	**0.01**	**< .001**	**0.10**	**0.01**	**< .001**
Right	**-0.65**	**0.01**	**< .001**	**-0.10**	**0.01**	**< .001**
Lighting source						
Left	**-2.49**	**0.34**	**< .001**	**-0.72**	**0.28**	**.010**
Right	**-2.69**	**0.35**	**< .001**	**-0.99**	**0.28**	**< .001**
Country (Japan)	**-0.70**	**0.29**	**.014**	**-0.82**	**0.23**	**< .001**

Notes. The reference level was specified as the ideal combination of the conditions (left-right offset: Center, distance: Close, facial rotation: Upright, lighting source: Front) and the country (Ireland) for convenience. The bold values indicate significant effects ($p < .05$).

Table S3

The proportion of correct prediction of gaze direction by iCatcher+ for the Non-anonymized dataset

	Modeled monitor size								
	Small			Medium			Large		
Term	Est.	SE	*p*-val.	Est.	SE	*p*-val.	Est.	SE	*p*-val.
Intercept	**-0.14**	**0.06**	**.01**	**0.28**	**0.04**	**< .001**	**0.66**	**0.13**	**< .001**
Left-right offset									
Left	**0.07**	**0.01**	**< .001**	-0.01	0.01	.21	**-0.16**	**0.01**	**< .001**
Right	-0.01	0.01	.34	**-0.08**	**0.01**	**< .001**	**-0.24**	**0.01**	**< .001**
Distance									
Middle	**-0.18**	**0.01**	**< .001**	**-0.22**	**0.01**	**< .001**	**-0.12**	**0.01**	**< .001**
Far	**-0.12**	**0.01**	**< .001**	**-0.30**	**0.01**	**< .001**	**-0.44**	**0.01**	**< .001**
Facial rotation									
Left	**-0.16**	**0.01**	**< .001**	**-0.20**	**0.01**	**< .001**	**-0.16**	**0.01**	**< .001**
Right	**-0.07**	**0.01**	**< .001**	**-0.30**	**0.01**	**< .001**	**-0.70**	**0.01**	**< .001**
Lighting source									
Left	**-0.53**	**0.07**	**< .001**	**-0.38**	**0.05**	**< .001**	0.14	0.16	.39
Right	**-0.43**	**0.07**	**< .001**	**-0.34**	**0.05**	**< .001**	0.08	0.16	.64
Country (Japan)	**0.22**	**0.06**	**< .001**	-0.01	0.04	.79	**-0.57**	**0.13**	**< .001**

Notes. The reference level was specified as the ideal combination of the conditions (left-right offset: Center, distance: Close, facial rotation: Upright, lighting source: Front) and the country (Ireland) for convenience. The bold values indicate significant effects ($p < .05$).

Table S4

The proportion of correct prediction of gaze direction by iCatcher+ for the Anonymized dataset

	Modeled monitor size								
	Small			Medium			Large		
Term	Est.	SE	*p*-val.	Est.	SE	*p*-val.	Est.	SE	*p*-val.
Intercept	**-4.73**	**0.06**	**< .001**	0.02	0.03	.53	**0.70**	**0.12**	**< .001**
Left-right offset									
Left	**0.03**	**0.01**	**< .001**	**0.04**	**0.01**	**< .001**	**-0.19**	**0.01**	**< .001**
Right	**0.02**	**0.01**	**.002**	**-0.07**	**0.01**	**< .001**	**-0.22**	**0.01**	**< .001**
Distance									
Middle	**-0.12**	**0.01**	**< .001**	**-0.18**	**0.01**	**< .001**	**-0.17**	**0.01**	**< .001**
Far	**-0.07**	**0.01**	**< .001**	**-0.26**	**0.01**	**< .001**	**-0.42**	**0.01**	**< .001**
Facial rotation									
Left	**-0.17**	**0.01**	**< .001**	**-0.17**	**0.01**	**< .001**	**-0.10**	**0.01**	**< .001**
Right	-0.00	0.01	.98	**-0.17**	**0.01**	**< .001**	**-0.42**	**0.01**	**< .001**
Lighting source									
Left	**-0.26**	**0.07**	**< .001**	**-0.29**	**0.05**	**< .001**	**-0.29**	**0.14**	**.043**
Right	-0.04	0.07	.55	**-0.17**	**0.05**	**< .001**	**-0.38**	**0.14**	**< .008**
Country (Japan)	**0.13**	**0.06**	**.02**	0.01	0.04	.71	**-0.27**	**0.12**	**.022**

Notes. The specification is the same as Table S3.

Table S5

The proportion of correct prediction of gaze direction by OWLET for the Non-anonymized dataset

	Modeled monitor size								
	Small			Medium			Large		
Term	Est.	SE	*p*-val.	Est.	SE	*p*-val.	Est.	SE	*p*-val.
Intercept	**-0.59**	**0.07**	**.01**	-0.07	0.06	.26	**0.70**	**0.08**	**< .001**
Left-right offset									
Left	**-0.04**	**0.01**	**< .001**	**-0.08**	**0.01**	**< .001**	**-0.07**	**0.01**	**< .001**
Right	-0.01	0.01	.38	**-0.03**	**0.01**	**< .001**	-0.01	0.01	.22
Distance									
Middle	**-0.54**	**0.01**	**< .001**	**-0.37**	**0.01**	**< .001**	**0.04**	**0.01**	**< .001**
Far	**-0.75**	**0.01**	**< .001**	**-0.62**	**0.01**	**< .001**	**-0.35**	**0.01**	**< .001**
Facial rotation									
Left	**0.03**	**0.01**	**.001**	0.01	0.01	.16	**-0.02**	**0.01**	**.01**
Right	**0.20**	**0.01**	**< .001**	**-0.08**	**0.01**	**< .001**	**-0.58**	**0.01**	**< .001**
Lighting source									
Left	**-0.40**	**0.08**	**< .001**	**-0.33**	**0.08**	**< .001**	**-0.25**	**0.10**	**.008**
Right	**-0.45**	**0.08**	**< .001**	**-0.35**	**0.08**	**< .001**	**-0.22**	**0.10**	**.02**
Country (Japan)	0.05	0.07	.41	0.07	0.06	.25	0.08	0.08	.29

Notes. The specification is the same as Table S3.

Table S6

The proportion of correct prediction of gaze direction by OWLET for the Anonymized dataset

	Modeled monitor size								
	Small			Medium			Large		
Term	Est.	SE	*p*-val.	Est.	SE	*p*-val.	Est.	SE	*p*-val.
Intercept	**-0.54**	**0.04**	**< .001**	**-0.12**	**0.04**	**< .001**	**0.61**	**0.04**	**< .001**
Left-right offset									
Left	**-0.11**	**0.01**	**< .001**	**-0.07**	**0.01**	**< .001**	**-0.06**	**0.01**	**< .001**
Right	**-0.08**	**0.01**	**< .001**	**-0.04**	**0.01**	**< .001**	**-0.05**	**0.01**	**< .001**
Distance									
Middle	**-0.46**	**0.01**	**< .001**	**-0.24**	**0.01**	**< .001**	**0.20**	**0.01**	**< .001**
Far	**-0.62**	**0.01**	**< .001**	**-0.44**	**0.01**	**< .001**	**-0.15**	**0.01**	**< .001**
Facial rotation									
Left	0.01	0.01	.09	0.00	0.01	.61	-0.01	0.01	.19
Right	**0.10**	**0.01**	**< .001**	**-0.07**	**0.01**	**< .001**	**-0.43**	**0.01**	**< .001**
Lighting source									
Left	**-0.27**	**0.05**	**< .001**	**-0.29**	**0.04**	**< .001**	**-0.34**	**0.05**	**< .001**
Right	**-0.34**	**0.05**	**< .001**	**-0.25**	**0.04**	**< .001**	**-0.14**	**0.05**	**.01**
Country (Japan)	**-0.03**	**0.04**	**.52**	-0.04	0.04	.24	-0.06	0.04	.13

Notes. The specification is the same as Table S3.

Figure S1

The proportion of the face detected by OWLET

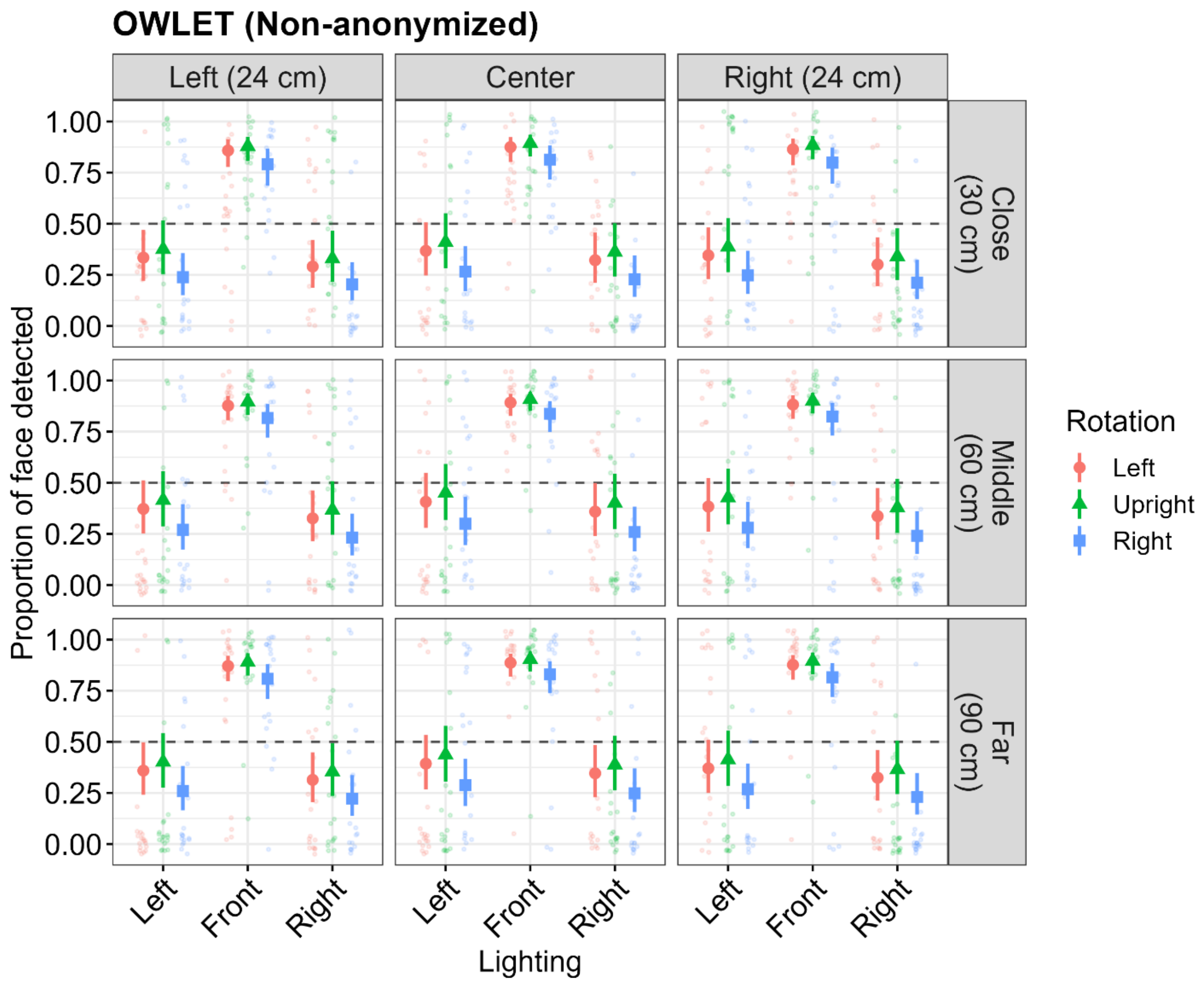

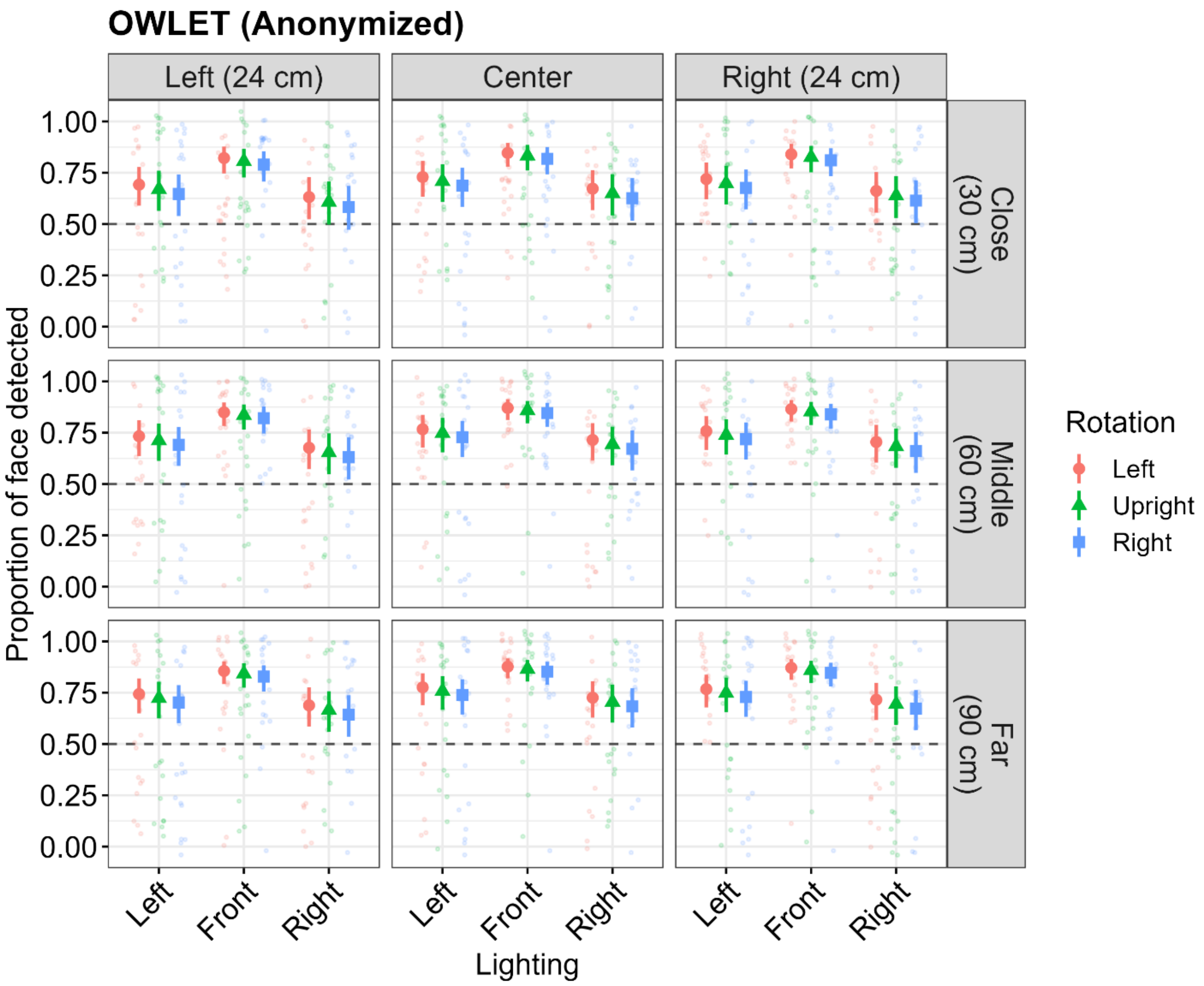

Notes. The colored points and their range represent the predicted values and 95% confidence intervals, respectively. The dashed horizontal line indicates the chance level. The translucent jittered points represent the mean proportion of face detection at each participant. In general, although face detection was negatively affected by the noise factors considered in this study (i.e., left-right offset, face rotation, lighting source, except for the distance to the webcam), faces were properly detected as long as the lighting source was located in front of the participants. This tendency was pronounced for the Non-anonymized dataset compared with the Anonymized dataset.

Figure S2

The proportion of correct prediction of gaze direction by iCatcher+ for the Non-anonymized dataset

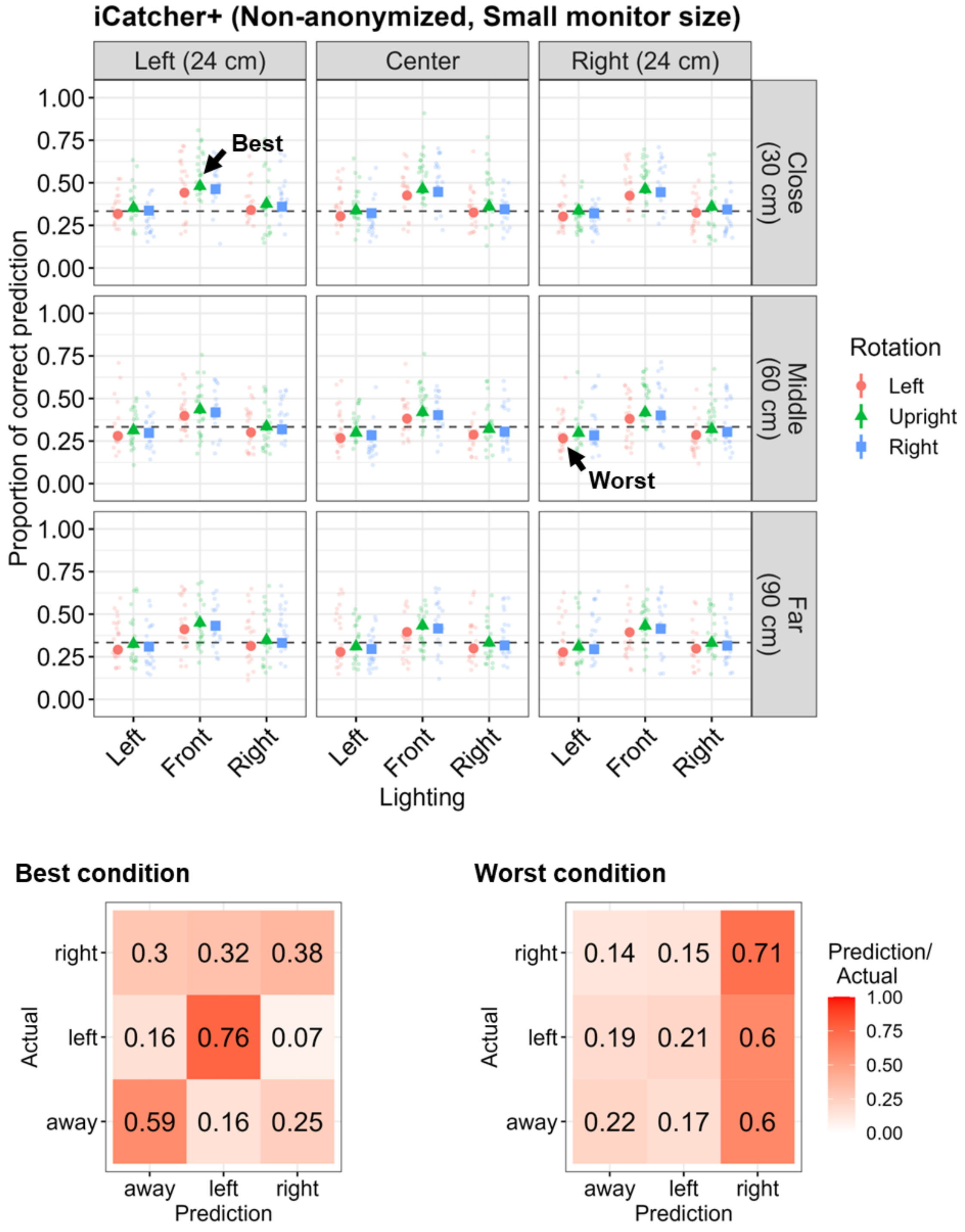

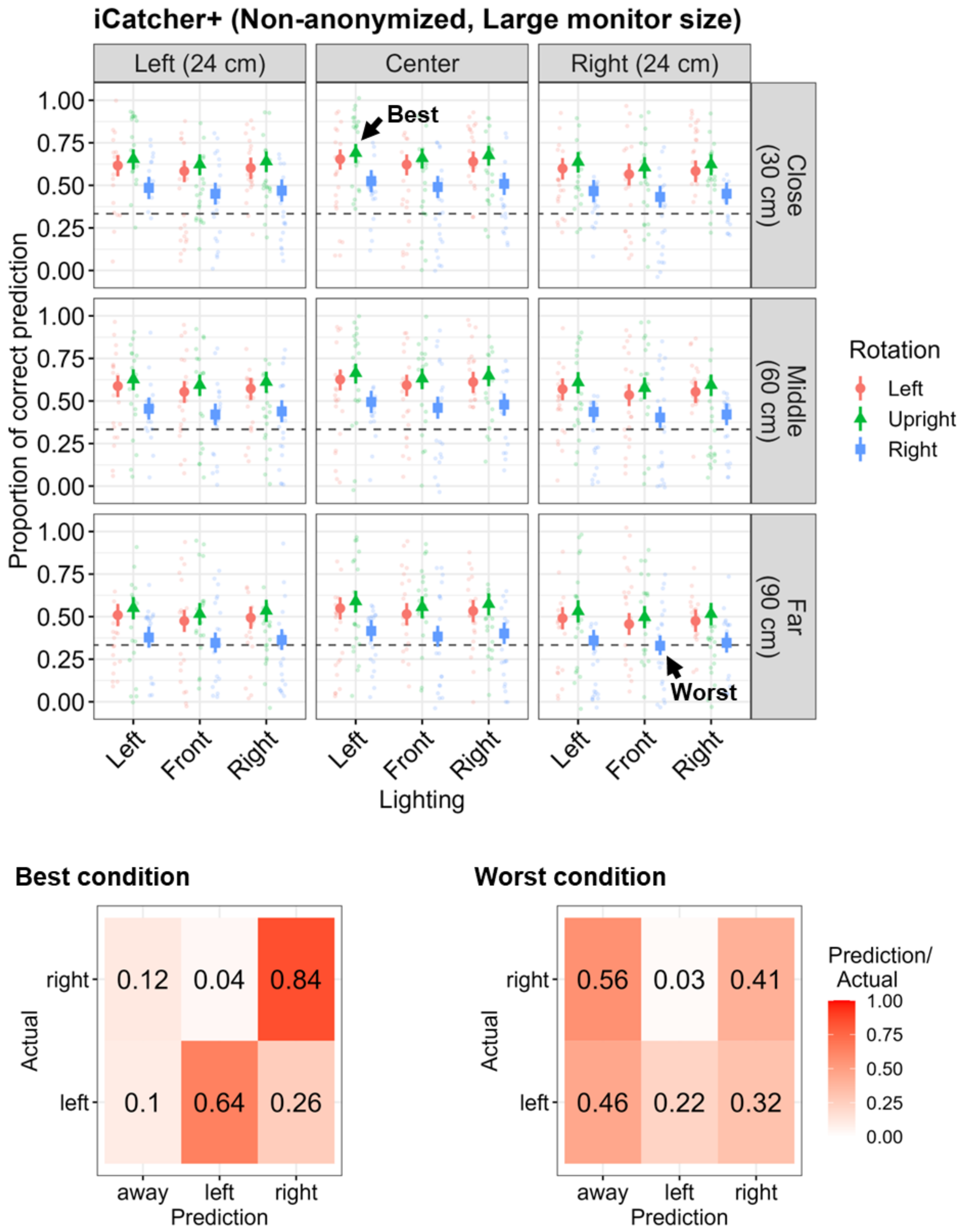

Notes. The specification is the same as Figure 3 in the main text.

Figure S3

The proportion of correct prediction of gaze direction by iCatcher+ for the Anonymized dataset

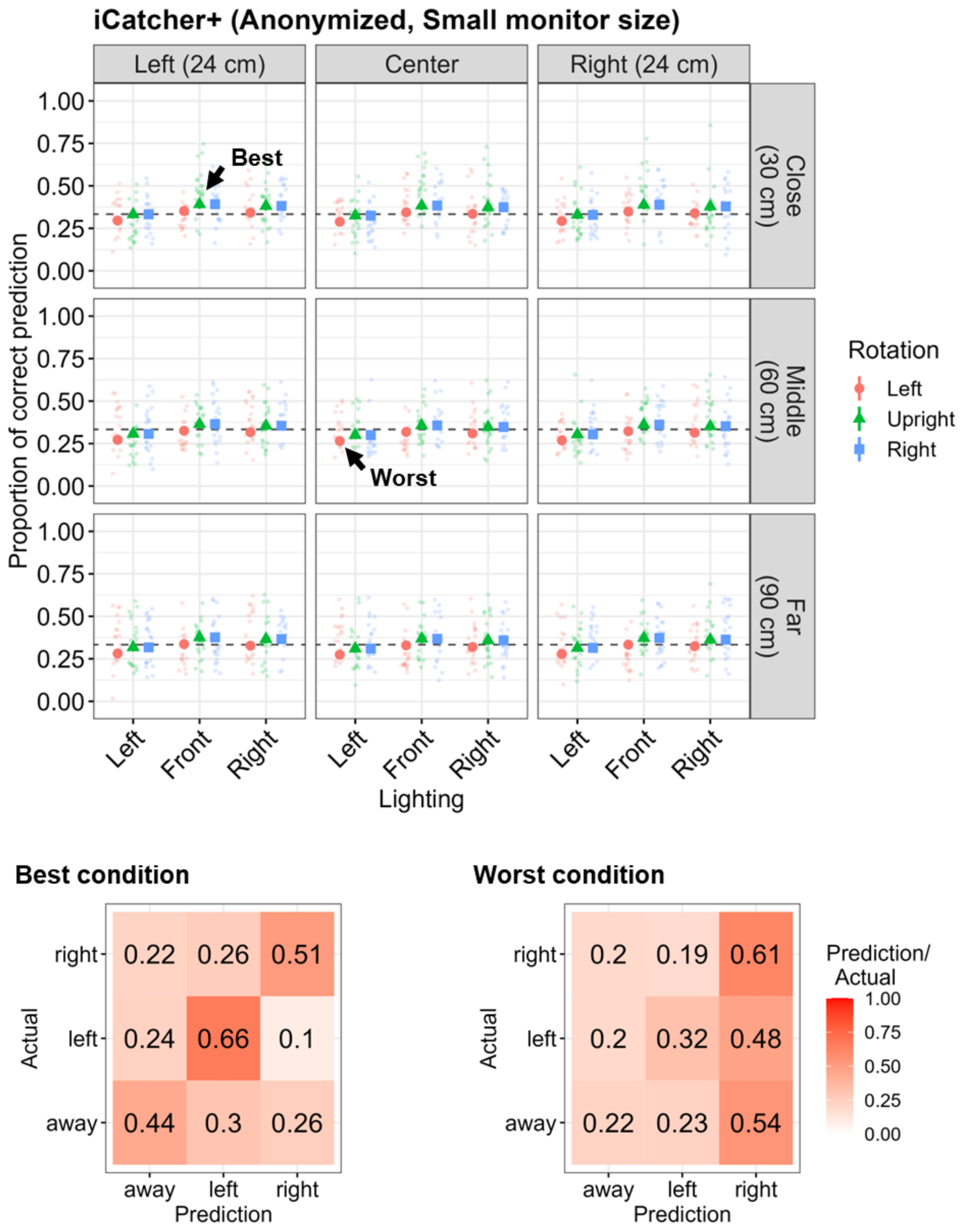

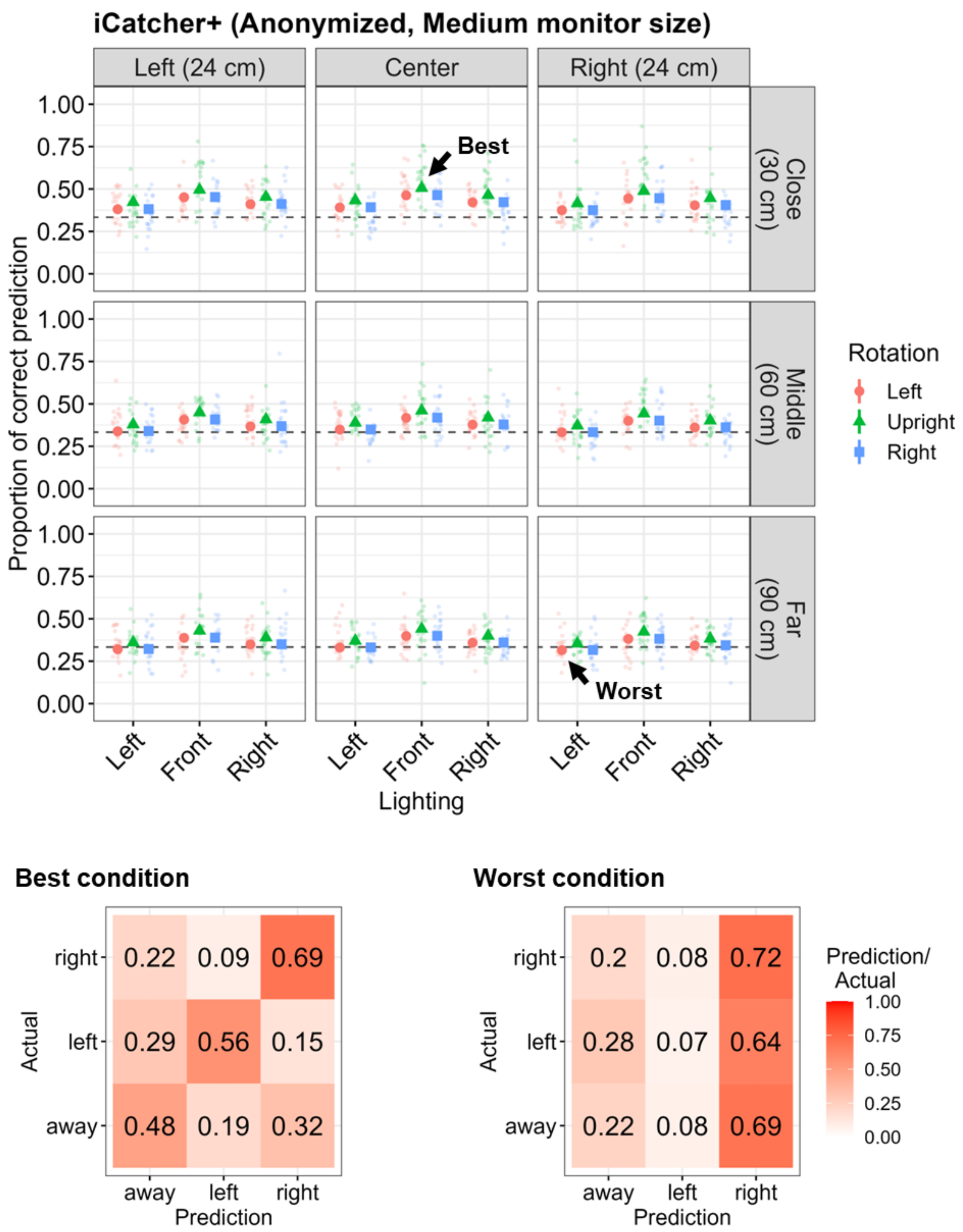
iCatcher+ (Anonymized, Medium monitor size)
Left (24 cm)
Center
Right (24 cm)
Close (30 cm)
Middle (60 cm)
Far (90 cm)
Proportion of correct prediction
1.00
0.75
0.50
0.25
0.00
Best
Worst
Left
Front
Right
Lighting
Rotation
Left
Upright
Right
Best condition
Worst condition
Actual
Prediction
right
left
away
0.22
0.09
0.69
0.29
0.56
0.15
0.48
0.19
0.32
0.2
0.08
0.72
0.28
0.07
0.64
0.22
0.08
0.69
Prediction/
Actual
1.00
0.75
0.50
0.25
0.00

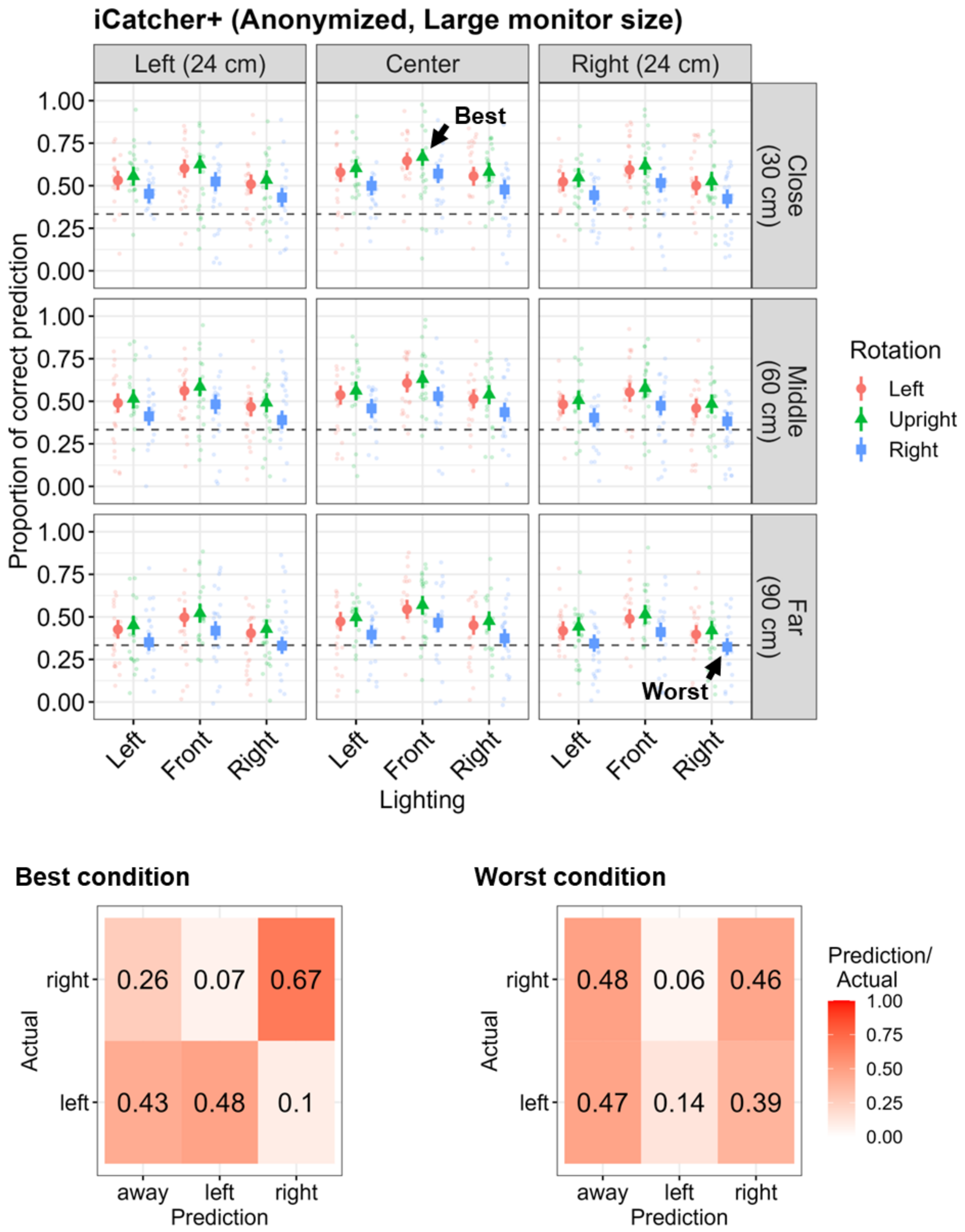

Notes. The specification is the same as Figure 3 in the main text.

Figure S4

The proportion of correct prediction of gaze direction by OWLET for the Non-anonymized dataset

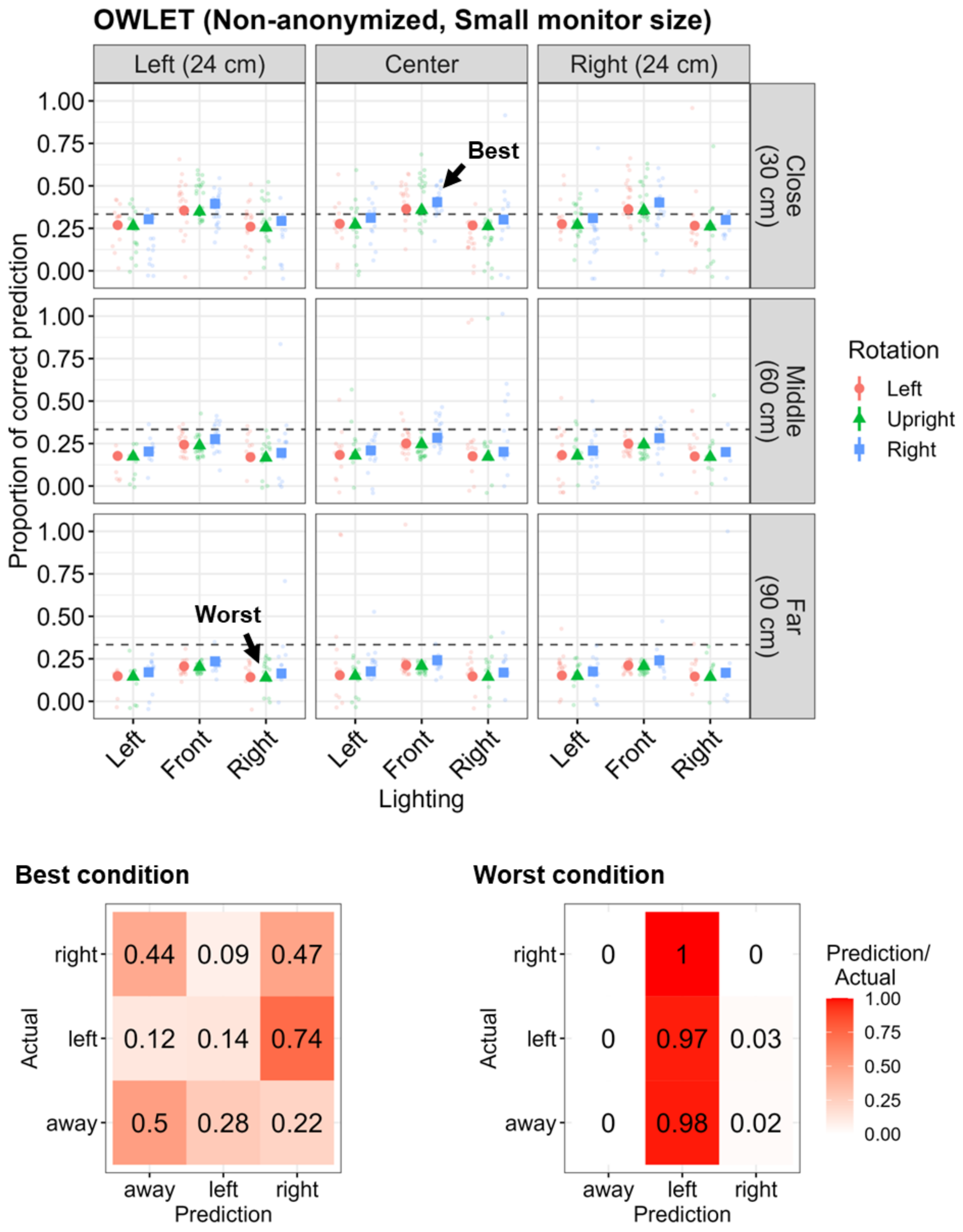

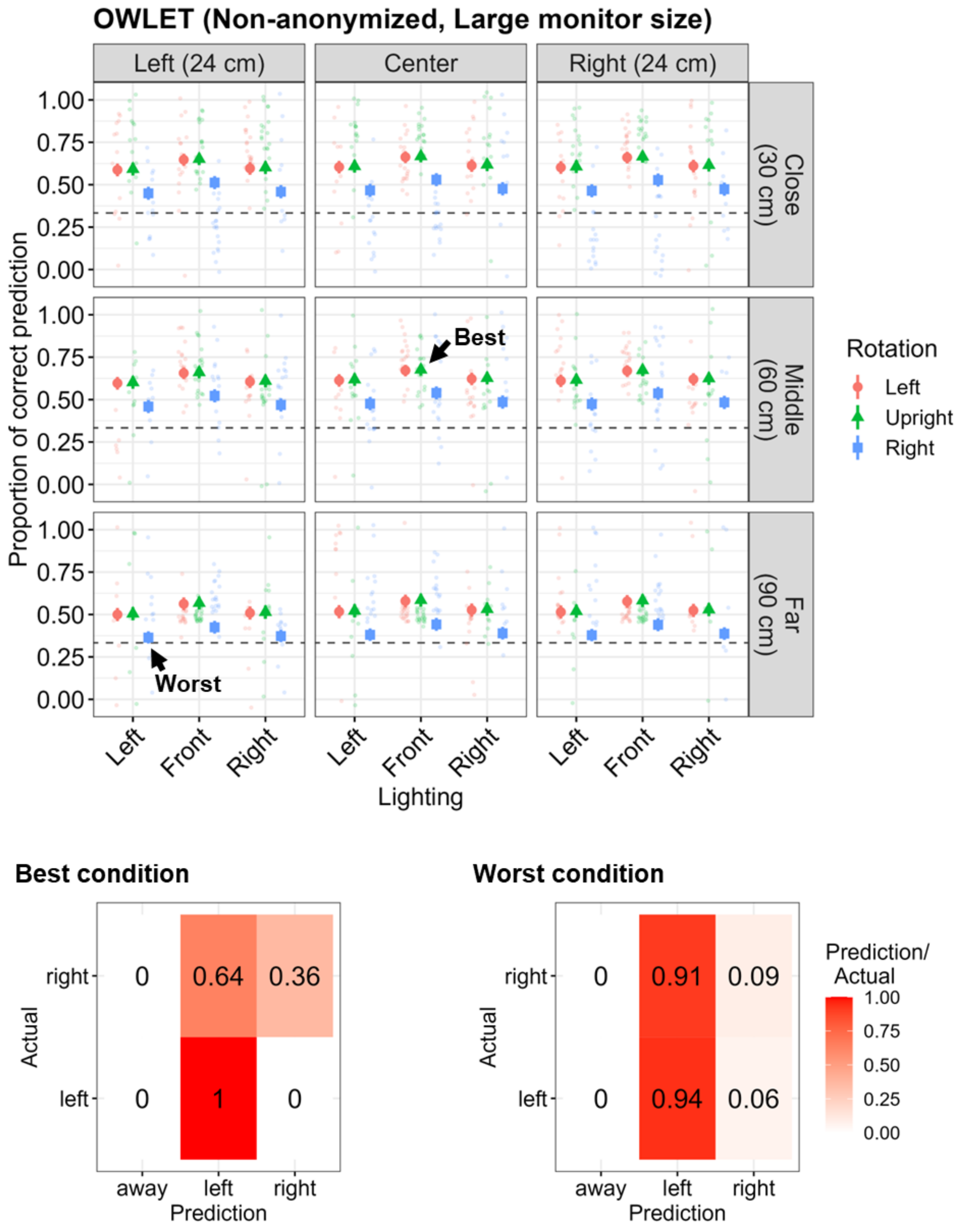

Notes. The specification is the same as Figure 3 in the main text.

Figure S5

The proportion of correct prediction of gaze direction by OWLET for the Anonymized dataset

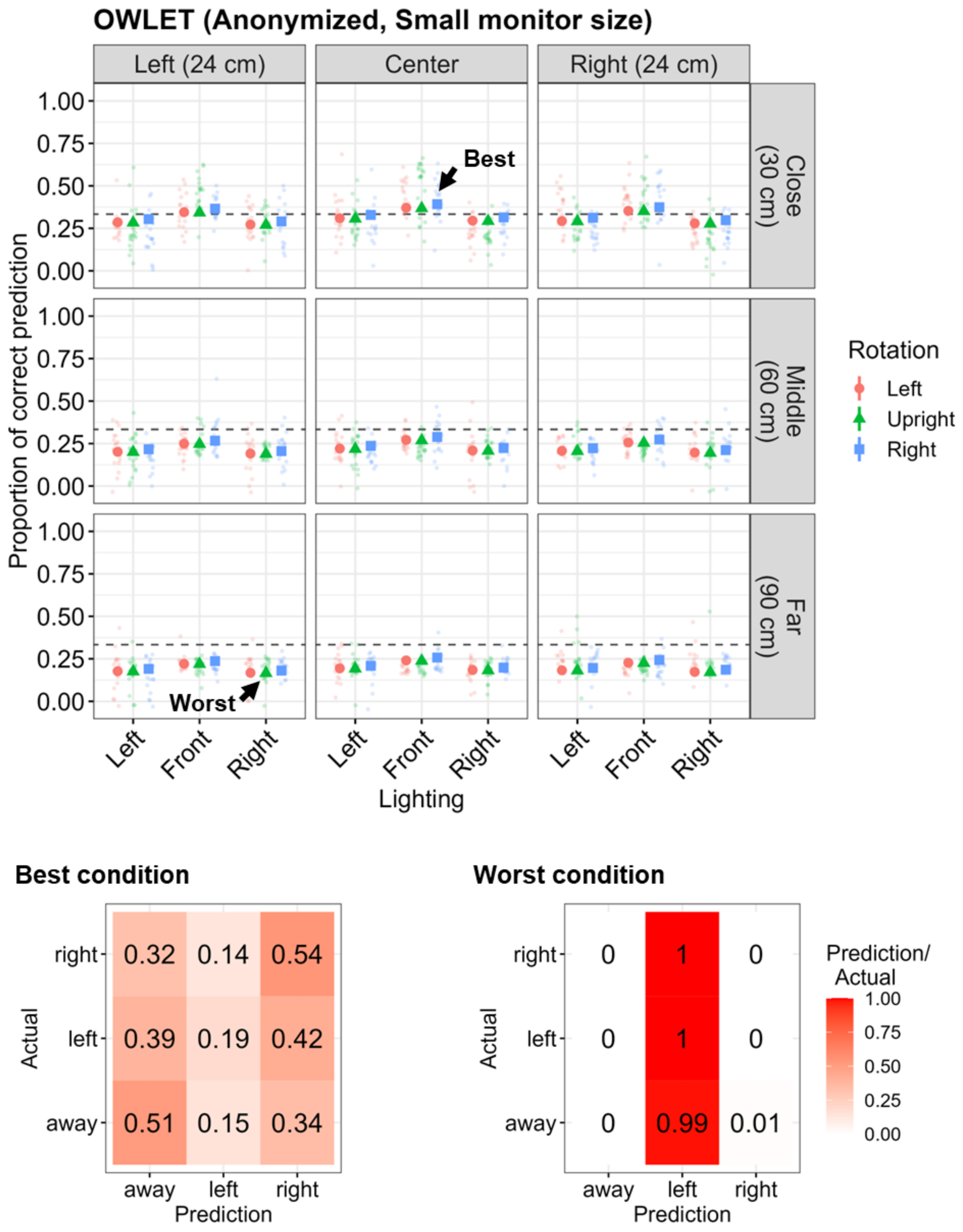

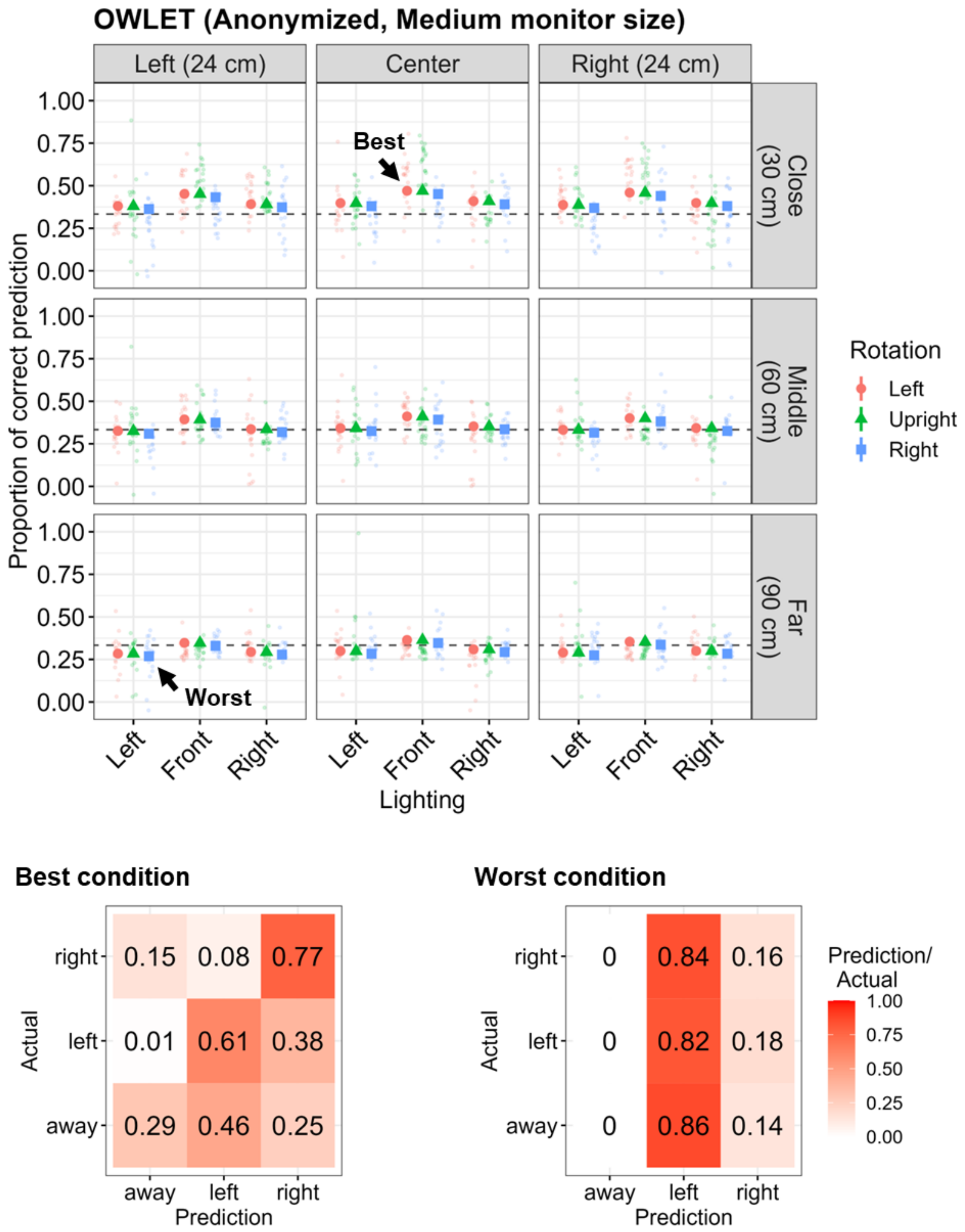
OWLET (Anonymized, Medium monitor size)
Left (24 cm)
Center
Right (24 cm)
Close (30 cm)
Middle (60 cm)
Far (90 cm)
Proportion of correct prediction
1.00
0.75
0.50
0.25
0.00
Best
Worst
Left
Front
Right
Lighting
Rotation
Left
Upright
Right
Best condition
Worst condition
Actual
Prediction
right
left
away
0.15 0.08 0.77
0.01 0.61 0.38
0.29 0.46 0.25
0 0.84 0.16
0 0.82 0.18
0 0.86 0.14
Prediction/ Actual

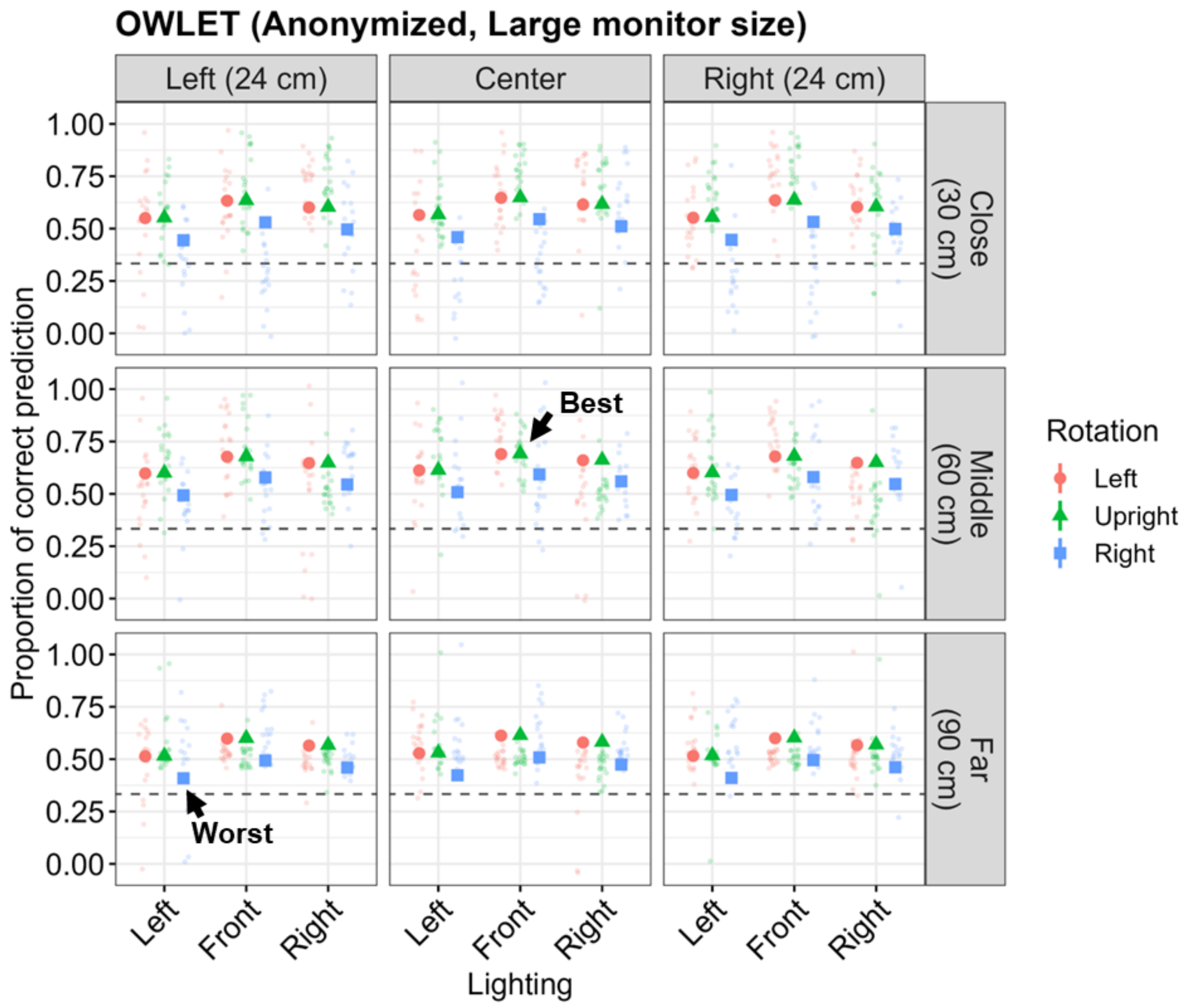

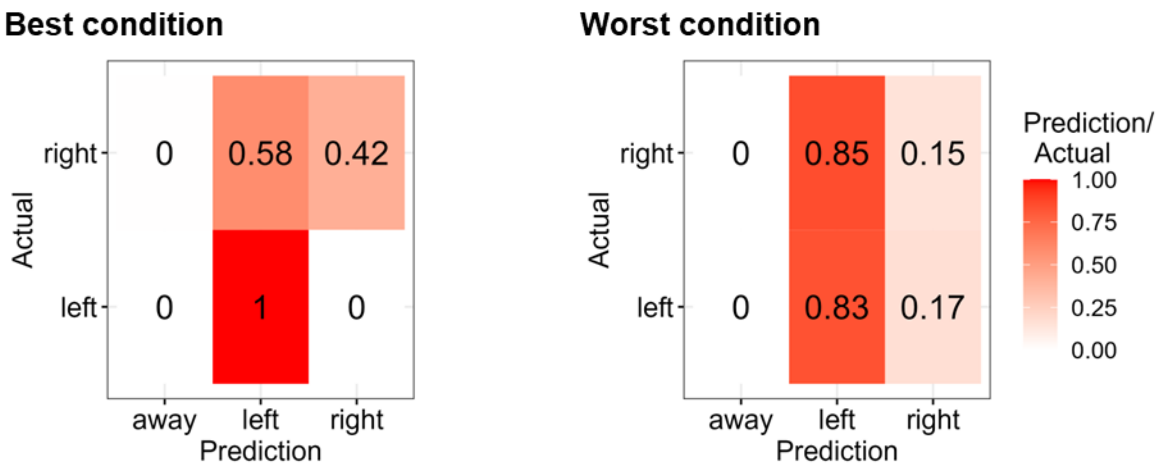

Notes. The specification is the same as Figure 3 in the main text.

Figure S6

The proportion of gaze direction estimations by iCatcher+ corresponding to each numbered disc

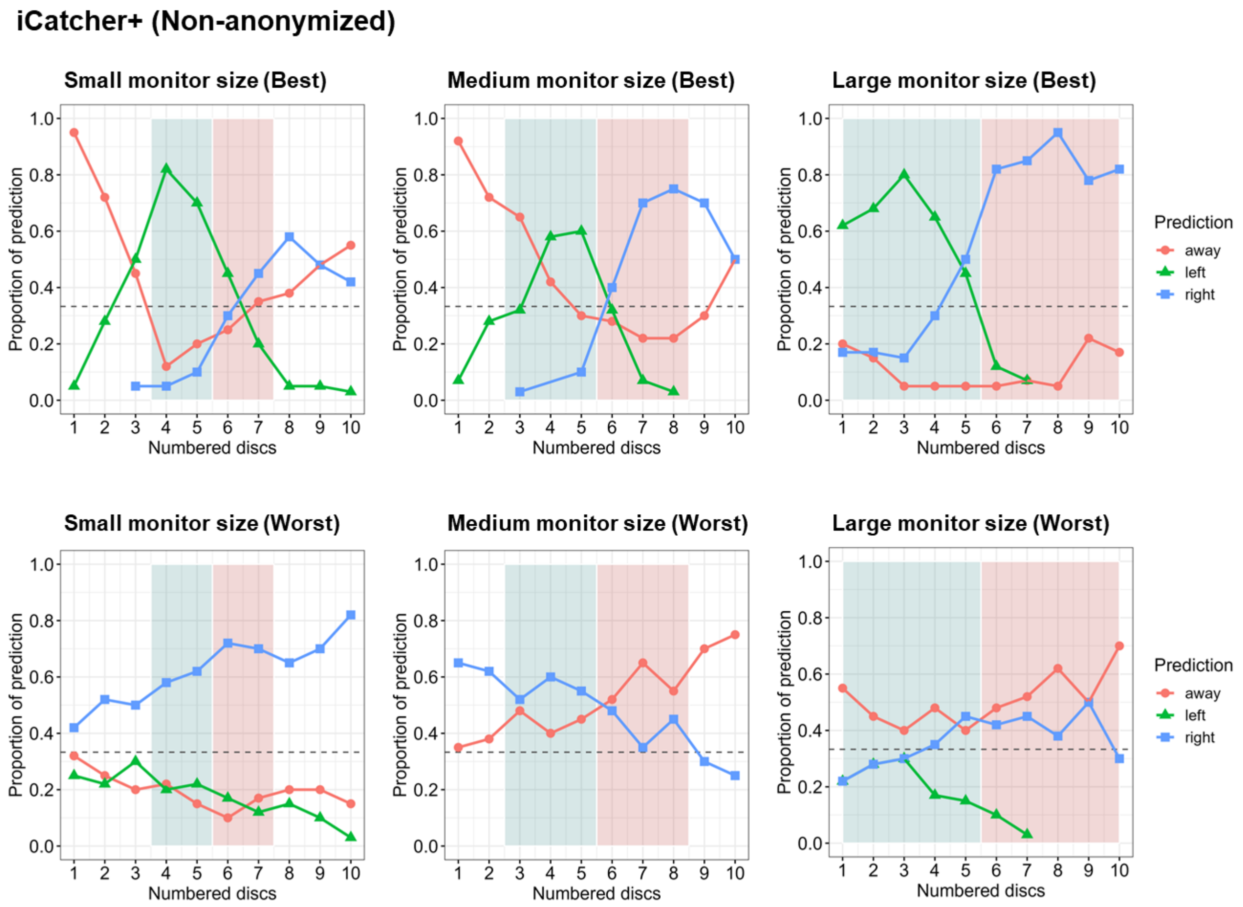

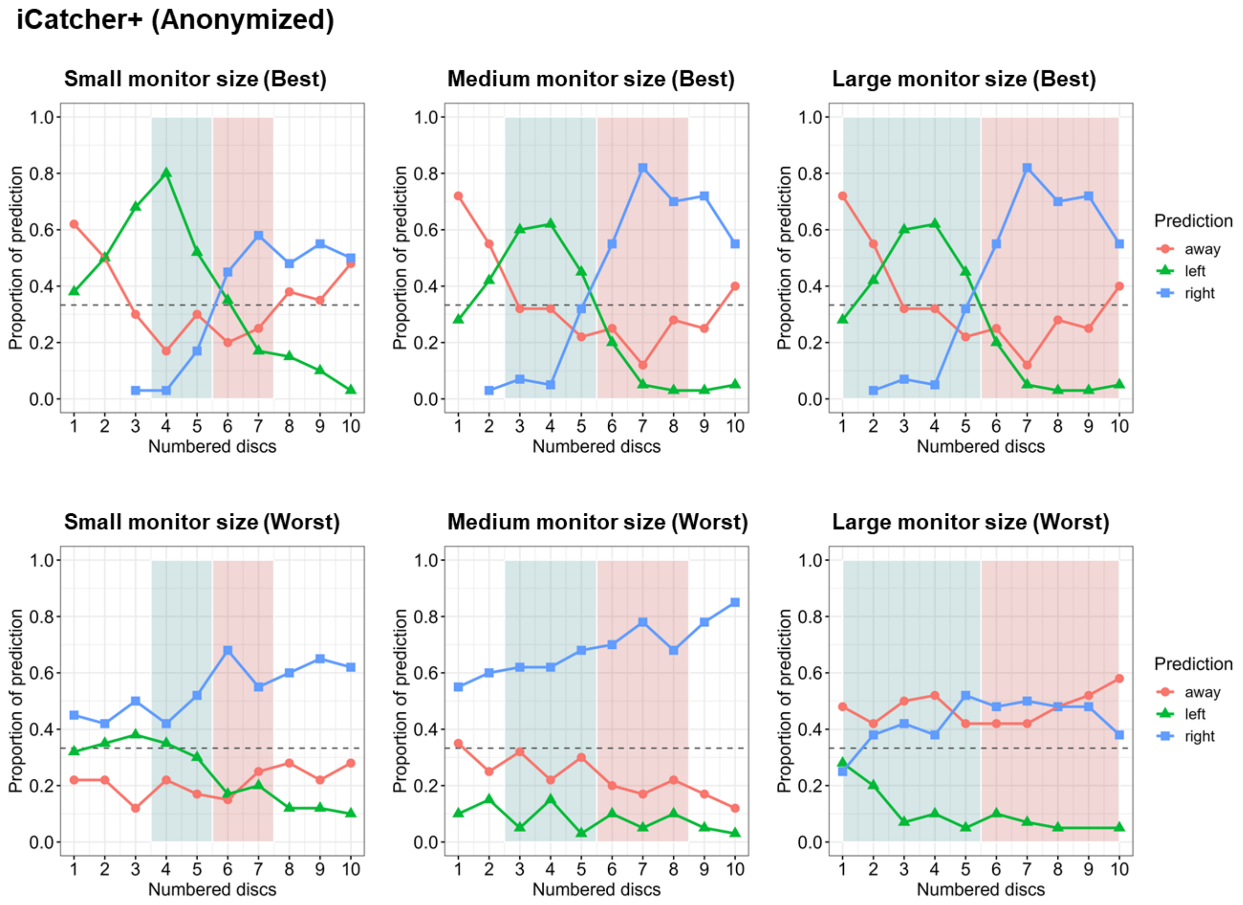

Notes. For each dataset and (modeled) monitor size, the shaded areas represent "within-monitor" (blue area = Left, red area = Right). The dashed lines represent the chance level.

Figure S7

The proportion of gaze direction estimations by OWLET corresponding to each numbered disc

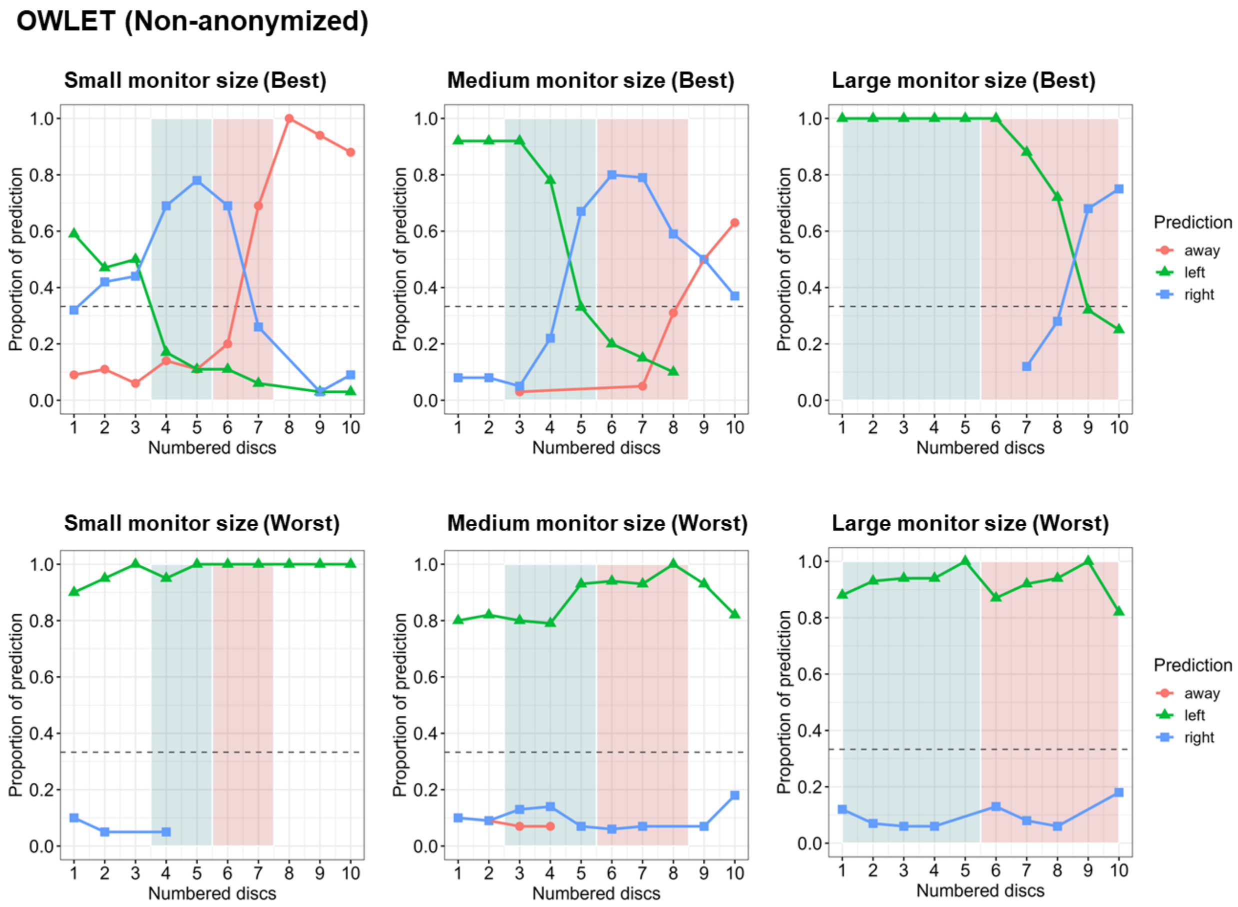

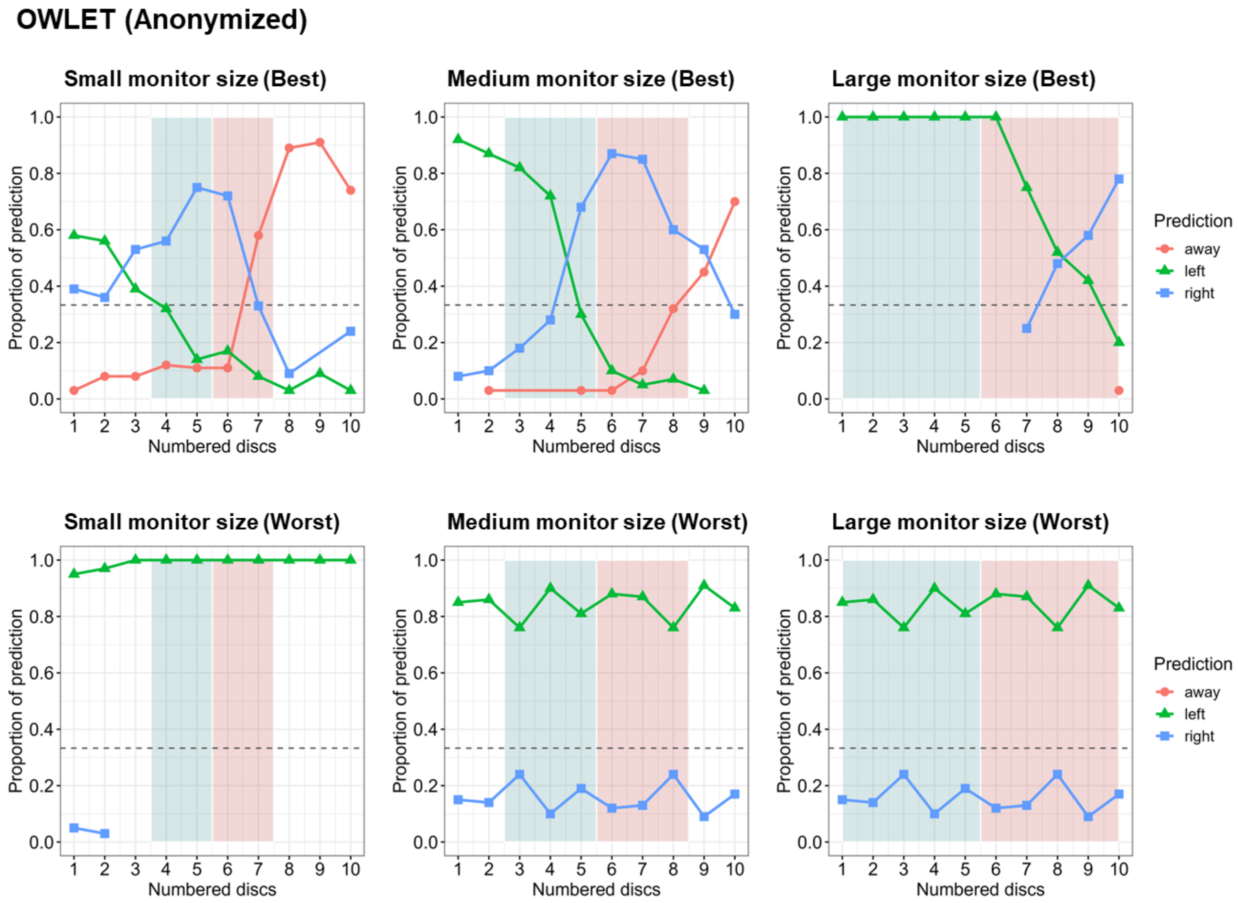

Notes. The specification is the same as Figure S6.
